# Computerized Cognitive Training in Cognitively Healthy Older Adults: A Systematic Review and Meta-Analysis of Effect Modifiers

**DOI:** 10.1371/journal.pmed.1001756

**Published:** 2014-11-18

**Authors:** Amit Lampit, Harry Hallock, Michael Valenzuela

**Affiliations:** Regenerative Neuroscience Group, Brain and Mind Research Institute, University of Sydney, Sydney, New South Wales, Australia; Mount Sinai School of Medicine, United States of America

## Abstract

**Background:**

New effective interventions to attenuate age-related cognitive decline are a global priority. Computerized cognitive training (CCT) is believed to be safe and can be inexpensive, but neither its efficacy in enhancing cognitive performance in healthy older adults nor the impact of design factors on such efficacy has been systematically analyzed. Our aim therefore was to quantitatively assess whether CCT programs can enhance cognition in healthy older adults, discriminate responsive from nonresponsive cognitive domains, and identify the most salient design factors.

**Methods and Findings:**

We systematically searched Medline, Embase, and PsycINFO for relevant studies from the databases' inception to 9 July 2014. Eligible studies were randomized controlled trials investigating the effects of ≥4 h of CCT on performance in neuropsychological tests in older adults without dementia or other cognitive impairment. Fifty-two studies encompassing 4,885 participants were eligible. Intervention designs varied considerably, but after removal of one outlier, heterogeneity across studies was small (*I* ^2^ = 29.92%). There was no systematic evidence of publication bias. The overall effect size (Hedges' *g*, random effects model) for CCT versus control was small and statistically significant, *g* = 0.22 (95% CI 0.15 to 0.29). Small to moderate effect sizes were found for nonverbal memory, *g* = 0.24 (95% CI 0.09 to 0.38); verbal memory, *g* = 0.08 (95% CI 0.01 to 0.15); working memory (WM), *g* = 0.22 (95% CI 0.09 to 0.35); processing speed, *g* = 0.31 (95% CI 0.11 to 0.50); and visuospatial skills, *g* = 0.30 (95% CI 0.07 to 0.54). No significant effects were found for executive functions and attention. Moderator analyses revealed that home-based administration was ineffective compared to group-based training, and that more than three training sessions per week was ineffective versus three or fewer. There was no evidence for the effectiveness of WM training, and only weak evidence for sessions less than 30 min. These results are limited to healthy older adults, and do not address the durability of training effects.

**Conclusions:**

CCT is modestly effective at improving cognitive performance in healthy older adults, but efficacy varies across cognitive domains and is largely determined by design choices. Unsupervised at-home training and training more than three times per week are specifically ineffective. Further research is required to enhance efficacy of the intervention.

*Please see later in the article for the Editors' Summary*

## Abstract 2

Michael Valenzuela and colleagues systematically review and meta-analyze the evidence that computerized cognitive training improves cognitive skills in older adults with normal cognition.

*Please see later in the article for the Editors' Summary*

## Editors' Summary

**Background:**

As we get older, we notice many bodily changes. Our hair goes grey, we develop new aches and pains, and getting out of bed in the morning takes longer than it did when we were young. Our brain may also show signs of aging. It may take us longer to learn new information, we may lose our keys more frequently, and we may forget people's names. Cognitive decline—developing worsened thinking, language, memory, understanding, and judgment—can be a normal part of aging, but it can also be an early sign of dementia, a group of brain disorders characterized by a severe, irreversible decline in cognitive functions. We know that age-related physical decline can be attenuated by keeping physically active; similarly, engaging in activities that stimulate the brain throughout life is thought to enhance cognition in later life and reduce the risk of age-related cognitive decline and dementia. Thus, having an active social life and doing challenging activities that stimulate both the brain and the body may help to stave off cognitive decline.

**Why Was This Study Done?:**

“Brain training” may be another way of keeping mentally fit. The sale of computerized cognitive training (CCT) packages, which provide standardized, cognitively challenging tasks designed to “exercise” various cognitive functions, is a lucrative and expanding business. But does CCT work? Given the rising global incidence of dementia, effective interventions that attenuate age-related cognitive decline are urgently needed. However, the impact of CCT on cognitive performance in older adults is unclear, and little is known about what makes a good CCT package. In this systematic review and meta-analysis, the researchers assess whether CCT programs improve cognitive test performance in cognitively healthy older adults and identify the aspects of cognition (cognitive domains) that are responsive to CCT, and the CCT design features that are most important in improving cognitive performance. A systematic review uses pre-defined criteria to identify all the research on a given topic; meta-analysis uses statistical methods to combine the results of several studies.

**What Did the Researchers Do and Find?:**

The researchers identified 51 trials that investigated the effects of more than four hours of CCT on nearly 5,000 cognitively healthy older adults by measuring several cognitive functions before and after CCT. Meta-analysis of these studies indicated that the overall effect size for CCT (compared to control individuals who did not participate in CCT) was small but statistically significant. An effect size quantifies the difference between two groups; a statistically significant result is a result that is unlikely to have occurred by chance. So, the meta-analysis suggests that CCT slightly increased overall cognitive function. Notably, CCT also had small to moderate significant effects on individual cognitive functions. For example, some CCT slightly improved nonverbal memory (the ability to remember visual images) and working memory (the ability to remember recent events; short-term memory). However, CCT had no significant effect on executive functions (cognitive processes involved in planning and judgment) or attention (selective concentration on one aspect of the environment). The design of CCT used in the different studies varied considerably, and “moderator” analyses revealed that home-based CCT was not effective, whereas center-based CCT was effective, and that training sessions undertaken more than three times a week were not effective. There was also some weak evidence suggesting that CCT sessions lasting less than 30 minutes may be ineffective. Finally, there was no evidence for the effectiveness of working memory training by itself (for example, programs that ask individuals to recall series of letters).

**What Do These Findings Mean?:**

These findings suggest that CCT produces small improvements in cognitive performance in cognitively healthy older adults but that the efficacy of CCT varies across cognitive domains and is largely determined by design aspects of CCT. The most important result was that “do-it-yourself” CCT at home did not produce improvements. Rather, the small improvements seen were in individuals supervised by a trainer in a center and undergoing sessions 1–3 times a week. Because only cognitively healthy older adults were enrolled in the studies considered in this systematic review and meta-analysis, these findings do not necessarily apply to cognitively impaired individuals. Moreover, because all the included studies measured cognitive function immediately after CCT, these findings provide no information about the durability of the effects of CCT or about how the effects of CCT on cognitive function translate into real-life outcomes for individuals such as independence and the long-term risk of dementia. The researchers call, therefore, for additional research into CCT, an intervention that might help to attenuate age-related cognitive decline and improve the quality of life for older individuals.

**Additional Information:**

Please access these websites via the online version of this summary at http://dx.doi.org/10.1371/journal.pmed.1001756.

- This study is further discussed in a *PLOS Medicine* Perspective by Druin Burch
- The US National Institute on Aging provides information for patients and carers about age-related forgetfulness, about memory and cognitive health, and about dementia (in English and Spanish)
- The UK National Health Service Choices website also provides information about dementia and about memory loss
- MedlinePlus provides links to additional resources about memory, mild cognitive impairment, and dementia (in English and Spanish)

## Introduction

Cognitive decline and impairment are amongst the most feared and costly aspects of aging [1]. The age-specific incidence of cognitive impairment is approximately double that of dementia [2],[3] and can be expected to affect 15%–25% of older individuals [2],[4]. Direct medical costs for older adults with mild cognitive impairment (MCI) are 44% higher than those for non-impaired older adults [5]. Because cognitive decline and impairment are essential criteria for dementia and often require informal care [5], interventions aimed at prevention or attenuation of such decline may have a substantial health and economic impact [3].

Several studies have now established strong and independent links between engagement in cognitively stimulating activities throughout the life span and enhanced late-life cognition, compression of cognitive burden, and reduced risk of cognitive impairment and dementia [6]–[8]. Intense interest has therefore focused on the potential of cognition-based interventions in older adults, especially computerized cognitive training (CCT) [9]. CCT involves structured practice on standardized and cognitively challenging tasks [10], and has several advantages over traditional drill and practice methods, including visually appealing interfaces, efficient and scalable delivery, and the ability to constantly adapt training content and difficulty to individual performance [9],[11]. Sales of commercial CCT packages may soon reach US$1 billion per year [12], but the evidence base for such products, at least in older adults, remains unclear [13].

Prior systematic reviews of generic cognitive interventions in healthy older adults [9],[14]–[18] have noted limitations, especially lack of supporting evidence from active-control trials and lack of replication due to inconsistent or indeterminate methodology. Importantly, these reviews pooled data from studies of CCT along with studies of other cognition-based interventions such as mnemonics or cognitive stimulation that can be as simple as reading newspapers or participating in group discussion [15]–[18]. It is therefore perhaps unsurprising that these reviews reached inconclusive results. A more recent systematic review in healthy older adults [9] was not restricted to randomized controlled trials (RCTs) and included CCT studies along with other computerized interventions such as classes in basic computer use.

The effectiveness of CCT in enhancing cognitive performance in healthy older adults is therefore currently unclear, and the impact of design and implementation factors on efficacy has yet to be systematically analyzed. Using data from RCTs of narrowly defined CCT, we aimed to quantitatively evaluate the efficacy of CCT with respect to multiple cognitive outcomes in healthy older adults. Furthermore, we aimed to test the moderating effect of several key study features in order to better inform future CCT trial design and clinical implementation.

## Methods

This work fully complies with the Preferred Reporting Items for Systematic Reviews and Meta-Analyses (PRISMA) guidelines [19] (see Checklist S1). Methods of analysis and inclusion criteria were specified in advance and are documented in Protocol S1.

### Eligibility Criteria

#### Types of studies

Eligible studies were published, peer-reviewed articles reporting results from RCTs of the effects of CCT on one or more cognitive outcomes in healthy older adults.

#### Types of participants

Eligible studies had mean participant age ≥60 y and participants who lacked any major cognitive, neurological, psychiatric, and/or sensory impairments. Studies with MCI as an inclusion criterion were excluded, as cognitive performance in this population may vary substantially, particularly with respect to variability in the diagnostic criteria of MCI [20].

#### Types of interventions

Eligible trials compared the effects of ≥4 h of practice on standardized computerized tasks or video games with clear cognitive rationale, administered on personal computers, mobile devices, or gaming consoles, versus an active or passive control condition. Lab-specific interventions that did not involve interaction with a computer were excluded.

#### Types of outcome measures

Outcomes included performance on one or more cognitive tests that were not included in the training program (i.e., untrained), administered both before and after training. This review is limited to change in performance from baseline to immediately post-training on tests of global cognition, verbal memory, nonverbal memory, working memory (WM), processing speed, attention, language, visuospatial skills, and executive functions. Both primary and secondary outcomes were included. Long-term outcomes, subjective measures (e.g., questionnaires), noncognitive outcomes (e.g., mood, physical), imaging data, and activities of daily living outcome measures were excluded from the analysis.

### Information Sources and Search Strategy

We searched Medline, Embase, and PsycINFO using the search terms “cognitive training” OR “brain training” OR “memory training” OR “attention training” OR “reasoning training” OR “computerized training” OR “computer training” OR “video game” OR “computer game”, and by scanning reference lists of previous reviews. No limits were applied for publication dates, and non-English papers were translated. The first search was conducted on 2 December 2013. An updated search was conducted on 9 July 2014.

### Study Selection

Two reviewers (A. L. and H. H.) independently screened search results for initial eligibility based on title and abstract. Full-text versions of potentially eligible studies and those whose eligibility was unclear based on title and abstract were assessed by A. L. and H. H., who also contacted authors when eligibility was unclear based on the full report. Disagreements regarding study eligibility were resolved by consulting with M. V., who approved the final list of included studies.

### Data Collection and Coding

Coding of outcome measures into cognitive domains was done by two reviewers (A. L. and H. H.) based on accepted neuropsychological categorization [21] or by consensus, and approved by M. V. Table S1 provides the coding of outcomes by cognitive domains. Data were entered into Comprehensive Meta-Analysis (CMA) version 2 (Biostat, Englewood, New Jersey). Data from most studies were entered as means and standard deviations (SDs) for the CCT and control groups at baseline and follow-up, with test–retest correlation set to 0.6. In a few instances, data were entered as post-training mean change [22]–[24] or raw mean difference with a 95% confidence interval [25]. CMA allows for each of these different study outcomes to be flexibly entered into the model. When data could not be extracted from study reports, we contacted the authors requesting raw summary data.

CCT programs were divided into five content types: speed of processing (SOP) training, WM training, attention training, multidomain training, and video games. Video games were defined as computer programs that were distributed for entertainment purposes before they were tried as cognitive interventions [26].

When studies presented data for both active and passive control groups, only the active control group was used as a comparison to the CCT group. When studies presented data from both young and older adults, only data from the older group were analyzed.

### Risk of Bias in Individual Studies and Study Appraisal

Risk of bias in individual studies was assessed using the items recommended in the Cochrane's Collaboration's risk of bias tool [27]: sequence generation; allocation concealment; blinding of participants, personnel, and outcome assessors; incomplete outcome data; selective outcome reporting; and other sources of bias. However, because the blinding of therapists and participants in CCT trials is impractical, we considered only blinding of assessors to determine risk of bias in the blinding item. We considered trials with high or unclear risk of bias those that did not include assessor blinding or did not perform intention-to-treat analyses. We considered all other trials as being at low risk of bias. Authors were contacted when the study details were unclear.

In addition, we used the Physiotherapy Evidence Database (PEDro) scale to assess study quality. The PEDro scale is a 11-item scale designed to assess the methodological quality and reporting of RCTs, and is reliable for rating trials of non-pharmacological interventions [28]. As with the risk of bias tool, we did not consider two items in the PEDro scale (blinding of therapists and participants), and therefore the maximum possible PEDro score for studies in this review was 9. All assessments were conducted by H. H. and additional external assessors (see Acknowledgments), and were subsequently reviewed by A. L.

### Data Analysis

The primary outcome was standardized mean difference (SMD, calculated as Hedges' *g*) of post-training change between CCT and control groups. Analyses were conducted for all cognitive results combined, as well as for each of the following cognitive domains: verbal memory, nonverbal memory, WM, processing speed, attention, visuospatial skills, and executive functions (planned analyses of global cognition and language were not performed because of insufficient numbers of studies reporting these outcomes). Precision of the SMD was calculated for each trial by the 95% CI. A positive SMD implies better therapeutic effects over time in the CCT group compared to the control group.

When studies presented data from more than one cognitive test, these were combined in two ways. First, all test results were combined to produce a single SMD per study, following established procedure [29]. Second, tests were classified on their main neuropsychological competency (see Table S1), such that each study could contribute to one or more cognitive-domain-specific SMDs. When outcomes from a given study were combined, the effect estimate was the mean amongst the related tests, and the estimate's variance was scaled up based on an assumed intercorrelation between the tests of 0.7 [30],[31]. All analyses were performed using CMA.

Because we expected studies to report multiple cognitive outcomes and display methodological variability [9],[13], our analyses were planned in three stages. First, in our main analysis we combined all outcomes from each study and pooled these to determine the overall efficacy of CCT in enhancing cognition. Second, we performed domain-specific meta-analyses, in which only studies that reported outcomes on a specified cognitive domain were included, using one combined SMD per study. Third, to examine between-study variability and identify design elements that may moderate observed efficacy, we performed subgroup meta-analyses. In the first and second stages, the overall and domain-specific meta-analyses were performed using a random-effects model. Using the same convention for description of Cohen's *d* effect sizes applied to Hedges' *g*, SMDs of ≤0.30, >0.30 and <0.60, and ≥0.60 were considered small, moderate, and large, respectively. Heterogeneity across studies was assessed using the *I* ^2^ statistic with 95% confidence (uncertainty) intervals [32],[33]. *I* ^2^ values of 25%, 50%, and 75% imply small, moderate, and large heterogeneity, respectively [33]. Forest plots were also used to visually characterize heterogeneity.

In the third stage, subgroup analyses were based on a mixed-effects model, which uses a random-effects model to generate within-subgroup variance and a fixed-effects model to compare effects between subgroups [34]. Between-subgroup heterogeneity was tested using the Cochrane's *Q* statistic [27] and was defined significant at the *p*<0.05 level. The following moderating factors were included in our analysis plan: type of CCT program (i.e., cognitive content of training), delivery format (group or home-based training), session length, session frequency, total duration of the program (dose), control condition (active or passive control), and risk of bias (high or low risk of bias as defined above).

### Risk of Bias across Studies

In order to assess risk of publication bias, funnel plots for overall outcomes as well as for each cognitive domain were inspected for asymmetry (i.e., SMDs charted against their standard error) [35]. When ten or more studies were pooled in a given meta-analysis, we formally tested funnel plot asymmetry using Egger's test of the intercepts [36]. A positive intercept implies that smaller studies tended to report more positive results than larger trials. When the test found notable asymmetry (*p*<0.1), we report primary outcomes based on a fixed-effects model along with a random-effects model, as the former gives more weight to larger trials and helps to counterbalance a possible inflation of therapeutic effect [35]; in these cases we discuss the more conservative effect estimate.

### Sensitivity Analyses

For the main analysis (efficacy across all cognitive outcomes), we tested the robustness of our results to parametric variation of the following assumptions: test–retest correlation (set at 0.6 and tested from 0.5 to 0.7), within-study multiple outcome intercorrelation (set at 0.7 and tested from 0.6 to 0.8), inclusion of passive controls instead of active controls in studies with multiple controls (*k* = 3), and use of a fixed-effects model instead of a random-effects model. These results are reported in Table S5.

## Results

### Study Selection

**Figure 1:**
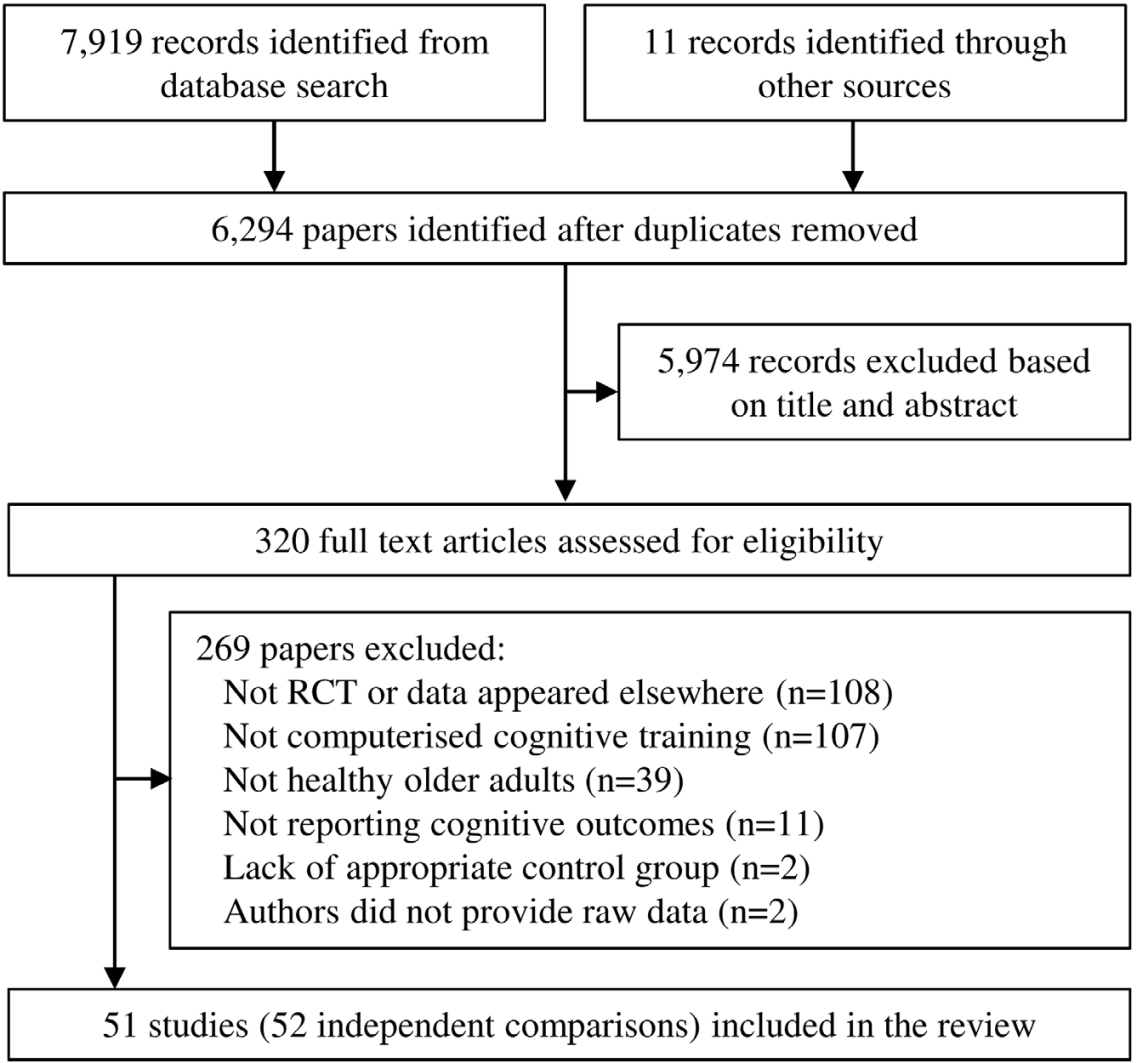
Summary of trial identification and selection. Note that a single study could be excluded on more than one criterion, but appears only once in the chart.

After duplicate search results were removed, 6,294 studies were initially screened for eligibility, of which 5,974 were excluded based on abstract and title. Three hundred twenty full-text articles were assessed for eligibility, of which 45 were deemed potentially eligible. After consulting with authors, three studies were excluded because they did not use randomized assignment [37]–[39], and a further two studies because authors did not provide necessary data [40],[41]. The resulting 40 studies from electronic search were supplemented by 11 studies [42]–[52] obtained by scanning reference lists of previous reviews and consulting with researchers, providing a total of 51 articles included in the analysis (Figure 1). Data from one article [53] were split into two studies, resulting in a final number of datasets cited in this review of 52 (for a detailed description of groups selected from each study, see Table S2).

We contacted 51 authors to request detailed summary data, enquire about possible eligibility, or determine risk of bias. Of these, 40 responded and provided information, two responded but did not provide information, and nine did not respond. Data for 14 studies were provided by authors [22],[23],[42],[49],[54]–[63] (see Table S3). The complete dataset is provided here as Dataset S1.

### Characteristics of Included Studies

Overall, the 52 datasets included in this review encompassed 4,885 participants (CCT, *n* = 2,527, mean group size = 49; controls *n* = 2,358, mean group size = 45; Table 1) and reported 396 cognitive outcomes. Mean participant age ranged from 60 to 82 y, and about 60% of participants were women. The cohorts were largely from the US [22],[25],[42],[45]–[47],[51]–[55],[61],[64]–[76] or Europe [23],[43],[44],[48],[56],[60],[63],[77]–[85], in addition to studies from Canada [57]–[59], Australia [49],[86], Israel [87], China [62], Taiwan Special Administrative Region [88], Republic of Korea [50], and Japan [24]. One study [49] was by authors of this review.

**Table 1:** Study characteristics.

Study	Study Demographics				Intervention Design							Study Design and Quality		
	*N*	Mean Age	Percent Female	MMSE Score	CCT Type	Delivery	Program	Dose^a^	Sessions^b^	Length^c^	Sessions/wk^d^	Control	Risk of Bias^e^	PEDro Score
Ackerman 2010 [64]	78	60.7	46.2		Multidomain	Home	Wii Big Brain Academy	20	20	60	5	Active	Low	4
Anderson 2013 [65]	67	63.0	58.2	27.4^f^	Multidomain	Home	PS Brain Fitness	40	40	60	5	Active	High	6
Anguera 2013 [22]	31	65.8	64.5	≥26	Attention	Home	In-house program	12	12	60	3	Passive	High	5
Ball 2002 [54]	1,398	73.6^g^	76^g^	27.3^g^	SOP	Group	SOP	11	10	67	2	Passive	Low	9
Barnes 2013 [42]	63	74.3	60.3	28.4^h^	Multidomain	Home	PS Brain Fitness+InSight	36	36	60	3	Active	Low	8
Basak 2008 [66]	34	69.6	74.4	29.3	Video game	Group	Rise of Nations	23.5	15	90	3	Passive	High	5
Belchior 2013 [53] study 1	27	74.3	40.7	29.1	Video game	Group	Medal of Honor	9	6	90	2–3	Passive	High	7
Belchior 2013 [53] study 2	31	74.7	61.3	29.3	SOP	Group	SOP	9	6	90	2–3	Active	High	7
Berry 2010 [55]	30	71.9	56.2	29.3	SOP	Mixed	PS Sweep Seeker	10	15	40	3–5	Passive	Low	6
Boot 2013 [67]	40	72.5	61	29	Multidomain	Home	Brain Age 2 (Nintendo DS)	60	60	60	5	Passive	High	7
Bottiroli 2009 [56]	44	66.2		27.6	Multidomain	Group	Neuropsychological training software	6	3	120	1	Passive	Low	7
Bozoki 2013 [68]	60	68.9	58.4	27.3^i^	Multidomain	Home	In-house program	30	30	60	5	Active	High	5
Brehmer 2012 [77]	45	63.8	60		WM	Home	Cogmed	9	23	26	4	Active	High	8
Burki 2014 [78]	45	68.1	76		WM	Group	In-house program	5	10	30	4	Passive	High	6
Buschkuehl 2008 [79]	39	80.0	59		Multidomain	Group	In-house program	18	24	45	2	Active	High	5
Casutt 2014 [80]	46	72.8	28.3		Attention	Group	In-house program	7	10	40	2	Passive	High	5
Colzato 2011 [43]	60	67.6	46.7	28.8	Multidomain	Home	In-house program	25	50	30	7	Active	High	5
Dahlin 2008 [44]	29	68.3	62.1	28.8	WM	Group	In-house program	11	15	45	3	Passive	High	5
Dustman 1992 [45]	40	66.3	60		Video game	Group	Various games	33	33	60	3	Active	High	6
Edwards 2002 [69]	97	73.7	56.7		SOP	Group	SOP	10	10	60	2	Passive	High	5
Edwards 2005 [47]	126	75.6		28.1	SOP	Group	SOP	10	10	60	2	Active	Low	7
Edwards 2013 [70]	60	73.1	69	28.1	Multidomain	Group	PS InSight	15	15	60	2–3	Passive	High	5
Garcia-Campuzano 2013 [57]	24	76.7	79.2		WM	Group	In-house program	12	24	30	3	Passive	Low	7
Goldstein 1997 [48]	22	77.7			Video game	Home	Tetris	26–37				Passive	High	5
Heinzel 2014 [81]	30	65.8	70	29.5	WM	Group	*n*-Back	9	12	45	3	Passive	High	6
Lampit 2014 [49]	77	72.1	68.8	28	Multidomain	Group	COGPACK	36	36	60	3	Active	Low	7
Lee 2012 [50]	30	73.8	53.3	27.0	SOP	Group	RehaCom	9	18	30	3	Active	High	4
Legault 2011 [71]	36	75.7	41.5	28.5^h^	WM	Group	In-house program	18	24	44	2	Active	High	7
Li 2010 [58]	20	76.2	65	26.9^f^	Attention	Group	Dual-task training	5	5	60	2	Passive	Low	8
Lussier 2012 [59]	23	68.5	78.3	28.5	Attention	Group	Dual-task training	5	5	60	2–3	Passive	High	7
Mahncke 2006 [46]	123	70.9^g^	50^g^	≥24	Multidomain	Home	PS Brain Fitness	40	40	60	5	Active	High	7
Maillot 2012 [23]	30	73.5	84.4	28.0	Multidomain	Group	Exergames (Nintendo Wii)	24	24	60	2	Passive	High	5
Mayas 2014 [60]	27	68.6	48.1	28.5	Multidomain	Group	Lumosity	20	20	60	2	Passive	High	3
McAvinue 2013 [82]	36	70.4	63.9	28.1	WM	Home	In-house program	14.75	25	35	5	Active	High	4
Miller 2013 [72]	69	81.9	67.7	28.0	Multidomain	Home	Dakim Brain Fitness	15	40	23	5	Passive	High	6
Nouchi 2012 [24]	28	69.1		28.5	Multidomain	Home	Brain Age (Nintendo DS)	5	20	15	5	Active	Low	7
O'Brien 2013 [61]	22	71.9	50	28.1	Multidomain	Group	PS InSight	17	14	70	2	Passive	Low	7
Peng 2012 [62]	50	70.4	80.8^g^		SOP	Group	Figure comparison	5	5	45–60	1	Passive	High	5
Peretz 2011 [87]	155	67.8	62	29.0	Multidomain	Home	CogniFit	16	36	25	3	Active	Low	8
Rasmusson 1999 [73]	24	79.2		27.8	Multidomain	Group	CNT	14	9	90	1	Passive	Low	7
Richmond 2011 [74]	40	66.0	80	29.0	WM	Home	In-house program	10	20	30	4	Active	High	6
Sandberg 2014 [83]	30	69.3	56.7	28.9	Multidomain	Group	In-house program	11	15	45	3	Passive	High	6
Shatil 2013 [75]	62	80.5	70	≥24	Multidomain	Group	CogniFit	32	48	40	3	Active	High	5
Shatil 2014 [84]	109	68.3	34.9	28.6	Multidomain	Group	CogniFit	8	24	20	3	Active	High	6
Simpson 2012 [86]	34	62.3	52.9	≥27	Multidomain	Home	MyBrainTrainer	7	21	20	7	Active	High	7
Smith 2009 [25]	487	75.3	52.4	29.2	Multidomain	Home	PS Brain Fitness	40	40	60	5	Active	Low	9
Stern 2011 [76]	40	66.7	54		Attention	Group	Space Fortress	36	36	60	3	Passive	High	7
van Muijden 2012 [63]	72	67.6	44.4	28.8	Multidomain	Home	In-house program	25	49	30	7	Active	High	6
Vance 2007 [51]	159	75.1	54.2	28.6	SOP	Group	SOP	10	10	60	1	Active	Low	5
von Bastian 2013 [85]	57	68.5	40.4	≥25	WM	Home	In-house program	16	20	27	5	Active	Low	7
Wang 2011 [88]	52	64.2	67.3	28.4	Attention	Group	In-house program	4	5	45	1	Passive	High	5
Wolinsky 2011 [52]	456	61.9	62.4		SOP	Group	PS On the Road	10	5	120	1	Active	Low	9

a Total number of training hours.

b Total number of CCT sessions.

c Session length (minutes).

d Number of sessions per week.

e Defined has having high or unclear risk of bias for blinding of assessors and/or incomplete outcome data.

f Measured with the Montreal Cognitive Assessment (MOCA, 1–30 scale).

g Means for the whole study (i.e., including groups that were not included in the analysis).

h Converted from the Modified Mental State Exam (3MSE, 1–100 scale) to Mini-Mental State Examination (1–30 scale).

i Measured with the St. Louis University Mental Status exam (SLUMS, 1–30 scale).

CNT, Colorado Neuropsychology Tests; MMSE, Mini-Mental State Examination; PS, Posit Science.

An active control group was used in 26 studies (50%), and assessor blinding was confirmed in 24 (46.2%) of studies. The average PEDro score was 6.2/9 (SD = 1.35), and 35 (66.6%) studies were found to have a high risk of bias (Table S4). As expected, risk of bias and study quality were connected: significant differences in PEDro scores were found for studies with high risk of bias (mean PEDro score = 5.69, SD = 1.08) compared to studies with low risk of bias (mean PEDro score = 7.18, SD = 1.33; *t*_(50)_ = −4.324, *p*<0.001).

Type of CCT varied considerably across studies (Table 1). Twenty-four studies used multidomain training, nine used SOP training, nine used WM training, six used attention training, and four were video games. Group (center-based) training was conducted in 32 (61.5%) of the studies, and 19 (36.5%) provided training at home. A study by Berry et al. [55] combined data from participants who trained at home with others who trained in research offices, and was therefore excluded from our subgroup analysis of delivery mode. In a study by Shatil et al. [84], 50 participants received group-based CCT and ten trained at home; data for the latter ten participants were excluded from the analysis (raw data for this study were provided in the online publication). Twenty-nine studies trained participants 2–3 times per week, 17 administered more than three sessions per week, and six administered only one session per week. Results of individual studies are provided in Table S2.

### Overall Efficacy on Cognitive Outcomes

**Figure 2:**
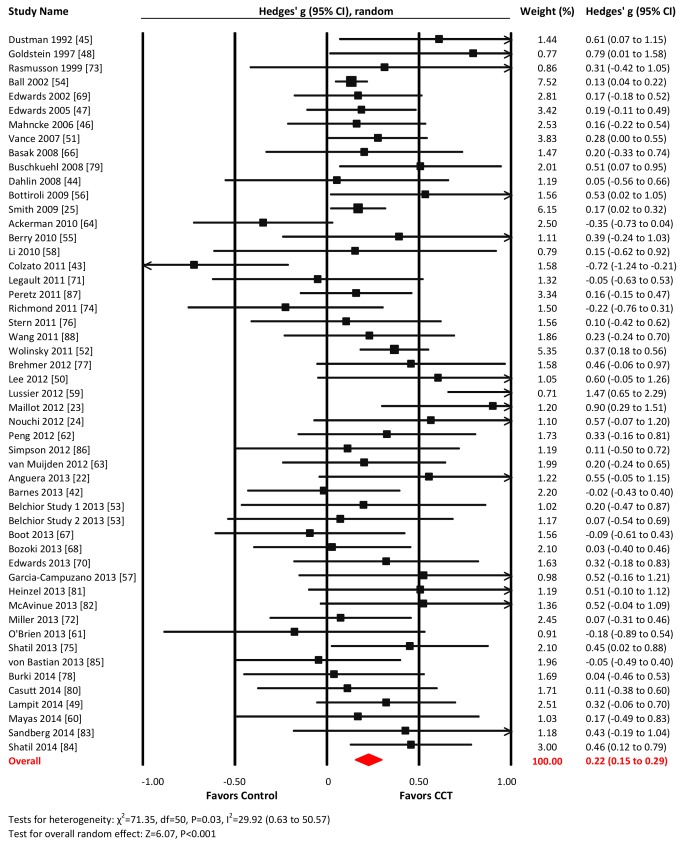
Overall efficacy of CCT on all cognitive outcomes. Effect estimates are based on a random-effects model, and studies are rank-ordered by year of publication.

**Figure 3:**
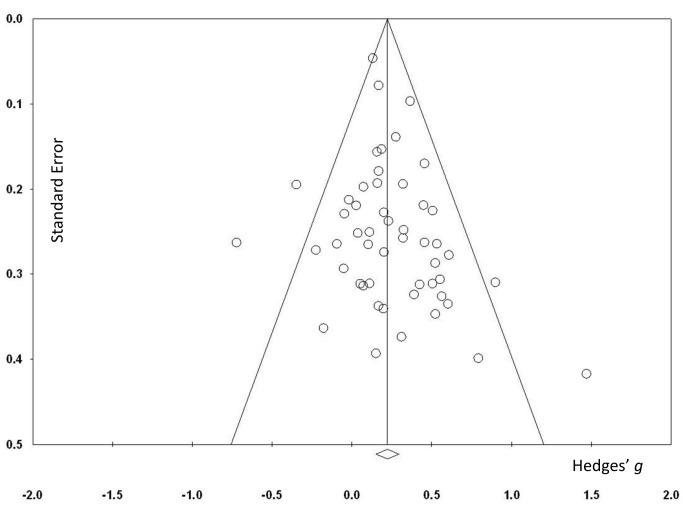
Funnel plot for overall effects after removal of one outlier [65].

The overall effect of CCT on cognition was small and statistically significant (*g* = 0.28, 95% CI 0.18 to 0.39, *p*<0.001). Heterogeneity across studies was moderate (*I* ^2^ = 69.03%, 95% CI 58.87% to 76.68%). The forest plot revealed one conspicuous outlier [65]: this study reported two extremely large SMDs (*g*>3.0; see Table S2) considered implausible and so was removed from all further analyses. Following this, heterogeneity reduced to a low level, and the summary effect size was reduced (*g* = 0.22, 95% CI 0.15 to 0.29, *p*<0.001; *I* ^2^ = 29.92%, 95% CI 0.63% to 50.57%; Figure 2). The resulting funnel plot did not show significant asymmetry (Egger's intercept = 0.48, *p* = 0.12; Figure 3). These results were robust to sensitivity analyses around our major assumptions (Table S5).

### Domain-Specific Efficacy

#### Verbal memory

**Figure 4:**
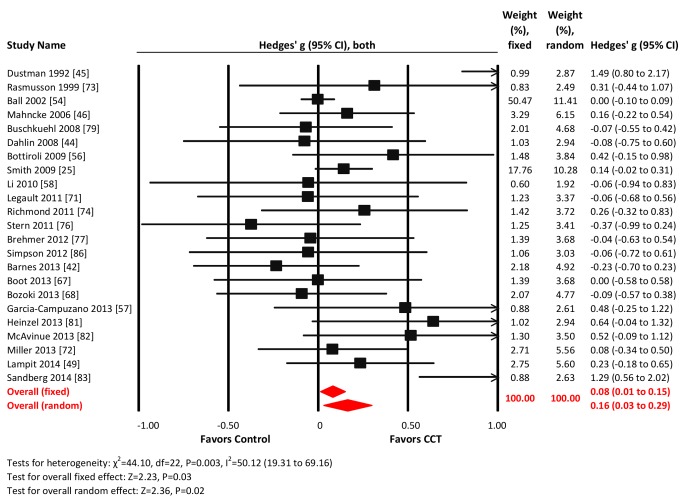
Efficacy of CCT on measures of verbal memory. Effect estimates are based on fixed-effects (top) and random-effects (bottom) models, and studies are rank-ordered by year of publication.

Twenty-three studies reported verbal memory outcomes. The combined effect size was small and statistically significant (*g* = 0.16, 95% CI 0.03 to 0.29, *p* = 0.02; Figure 4). Heterogeneity across studies was moderate (*I* ^2^ = 50.12%, 95% CI 19.31% to 69.16%). The Funnel plot showed potential asymmetry (Egger's intercept = 0.81, *p* = 0.07; Figure S1). A fixed-effects analysis was therefore conducted and revealed a very small effect size (*g* = 0.08, 95% CI 0.01 to 0.15, *p* = 0.03; Figure 4).

#### Nonverbal memory

**Figure 5:**
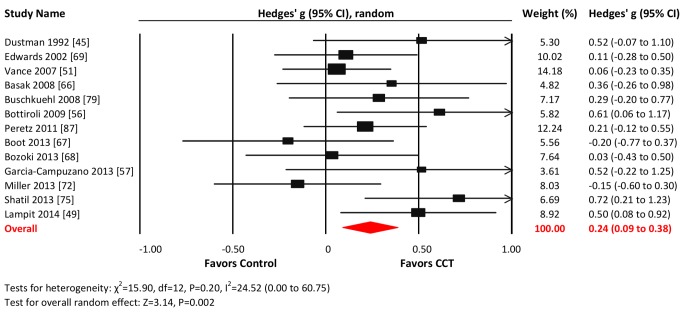
Efficacy of CCT on measures of nonverbal memory. Effect estimates are based on a random-effects model, and studies are rank-ordered by year of publication.

Thirteen studies reported nonverbal memory outcomes. The combined effect size was small and statistically significant (*g* = 0.24, 95% CI 0.09 to 0.38, *p* = 0.002; Figure 5). Heterogeneity across studies was small (*I* ^2^ = 24.52%, 95% CI 0% to 60.75%), and the funnel plot did not show evidence of asymmetry (Egger's intercept = 1.75 *p* = 0.18; Figure S1).

#### Working memory

**Figure 6:**
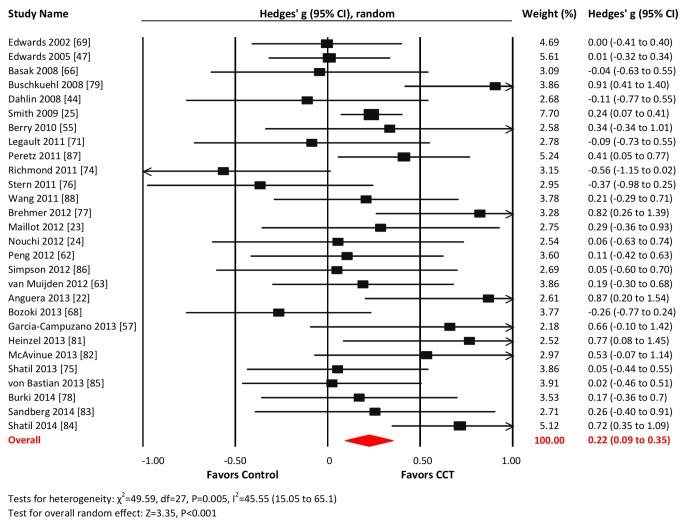
Efficacy of CCT on measures of working memory. Effect estimates are based on a random-effects model, and studies are rank-ordered by year of publication.

Twenty-eight studies reported WM outcomes. The combined effect size was small and statistically significant (*g* = 0.22, 95% CI 0.09 to 0.35, *p*<0.001; Figure 6). Heterogeneity across studies was moderate (*I* ^2^ = 45.55%, 95% CI 15.05% to 65.1%). The funnel plot did not show significant asymmetry (Egger's intercept = −0.1, *p* = 0.89; Figure S1).

#### Processing speed

**Figure 7:**
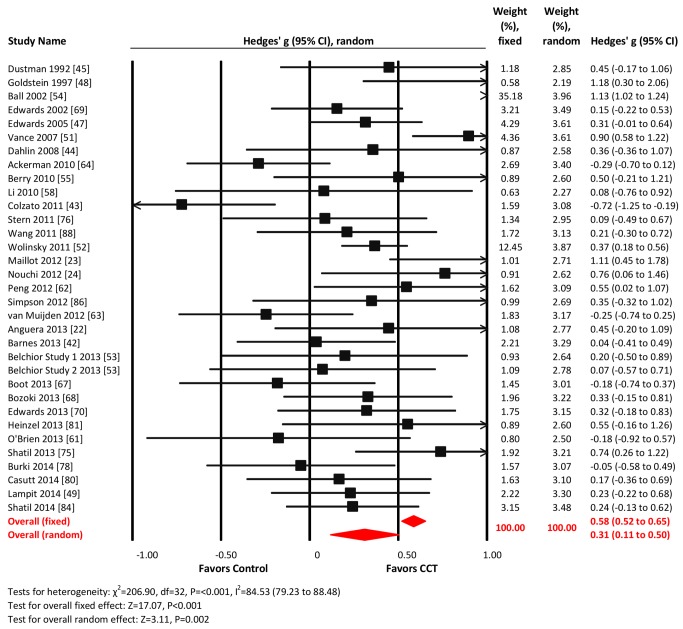
Efficacy of CCT on measures of processing speed. Effect estimates are based on fixed-effects (top) and random-effects (bottom) models, and studies are rank-ordered by year of publication.

Thirty-three studies reported processing speed outcomes. The combined effect size was moderate and statistically significant (*g* = 0.31, 95% CI 0.11 to 0.50, *p* = 0.002; Figure 7). Heterogeneity across studies was large (*I* ^2^ = 84.53%, 95% CI 79.23% to 88.48%). We detected evidence of unusual funnel plot asymmetry, whereby larger studies reported larger effect sizes (Egger's intercept = −2.99, *p*<0.01; Figure S1). A fixed-effects analysis revealed a substantially larger effect size (*g* = 0.58, 95% CI 0.52 to 0.65, *p*<0.001; Figure 4).

#### Executive functions

**Figure 8:**
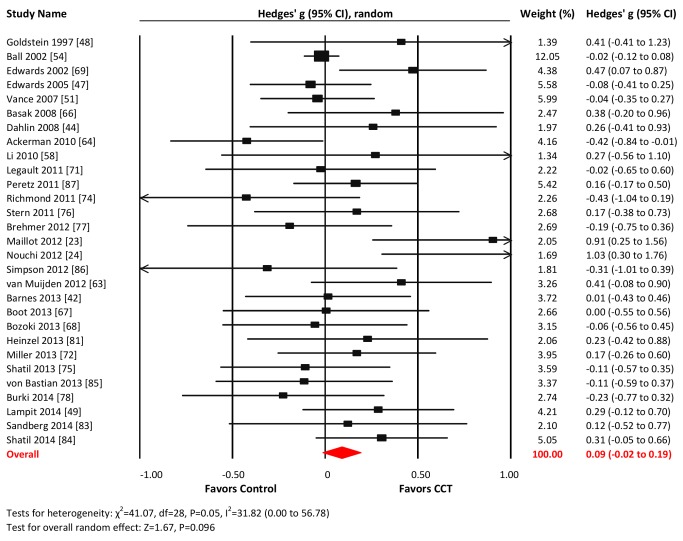
Efficacy of CCT on measures of executive functions. Effect estimates are based on a random-effects model, and studies are rank-ordered by year of publication.

Twenty-nine studies reported outcomes with measures of executive functions. The combined effect size was negligible and statistically non-significant (*g* = 0.09, 95% CI −0.02 to 0.19, *p* = 0.096; Figure 8). Heterogeneity across studies was small (*I* ^2^ = 31.82%, 95% CI 0% to 56.78%). The funnel plot suggested larger effect sizes in smaller studies (Egger's intercept = 0.65, *p* = 0.097; Figure S1).

#### Attention

**Figure 9:**
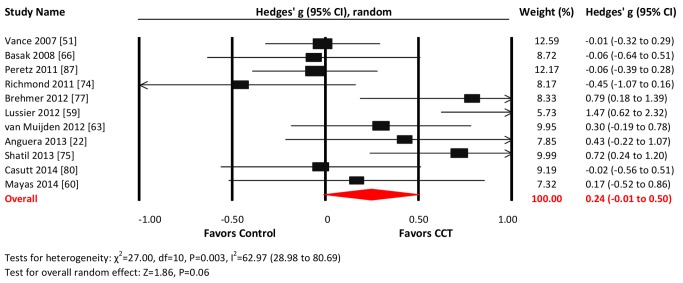
Efficacy of CCT on measures of attention. Effect estimates are based on a random-effects model, and studies are rank-ordered by year of publication.

Eleven studies reported attention-related outcomes. The combined effect size was small and non-significant (*g* = 0.24, 95% CI −0.01 to 0.50, *p* = 0.06; Figure 9). Heterogeneity across studies was moderate (*I* ^2^ = 62.97%, 95% CI 28.98% to 80.69%). The funnel plot did not display notable asymmetry (Egger's intercept = 2.61, *p* = 0.13; Figure S1).

#### Visuospatial skills

**Figure 10:**
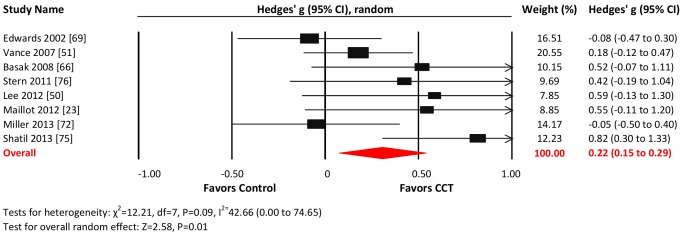
Efficacy of CCT on measures of visuospatial skills. Effect estimates are based on a random-effects model, and studies are rank-ordered by year of publication.

Eight studies reported visuospatial outcomes. The combined effect size was small and statistically significant (*g* = 0.22, 95% CI 0.15 to 0.29, *p* = 0.01; Figure 10). Heterogeneity across studies was moderate (*I* ^2^ = 42.66%, 95% CI 0% to 74.65%). The funnel plot revealed potential asymmetry, suggesting a greater effect in smaller studies (Figure S1), but formal testing was not conducted because of the small number of studies.

#### Global cognition and language

Planned analyses of global cognition and language were not performed as these outcomes were reported in only three studies each ([24],[50],[88] and [49],[72],[75], respectively).

### Moderators of CCT Efficacy

**Figure 11:**
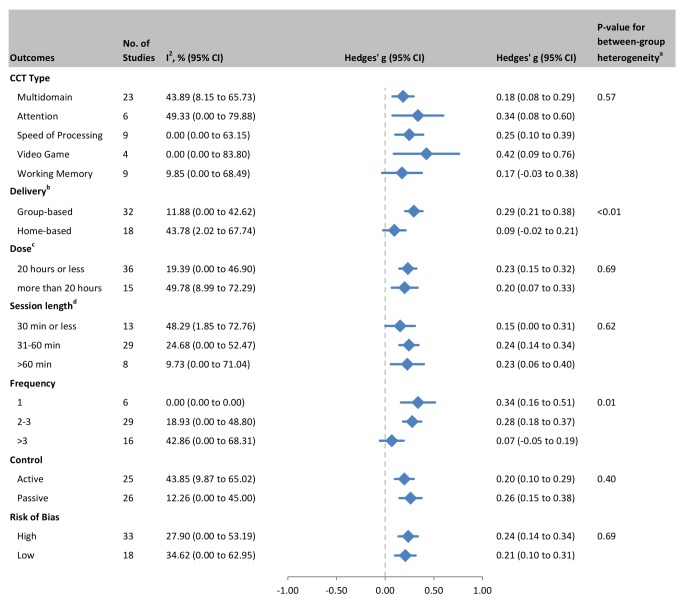
Subgroup analyses of moderators of overall efficacy of CCT in older adults. ^a^ *Q-*test for between-group heterogeneity, mixed-effects model. ^b^One study that combined data from both home- and group-based training [55] was excluded from this analysis. ^c^Total number of training hours. ^d^Session length could not be determined for one study.

In order to examine the relationship between CCT design choices and training outcomes, we evaluated efficacy in predefined subgroups (Figure 11). Based on all cognitive outcomes, there was a significant difference in the efficacy of group-based training (*g* = 0.29, 95% CI 0.21 to 0.38, *p*<0.001) compared to home-based administration (*g* = 0.09, 95% CI −0.02 to 0.21, *p* = 0.11; *Q* statistic for between-group heterogeneity = 7.183, df = 1, *p* = 0.007). Study-to-study heterogeneity within the group-based training studies was low (*I^2^* = 11.88%, CI 0% to 43%; *Q* = 35.18, df = 31, *p* = 0.28; Figure 11). There was also a significant effect for training frequency, with significant effect estimates in studies that administered one (*g* = 0.34, 95% CI 0.16 to 0.51, *p*<0.001) or 2–3 sessions per week (*g* = 0.28, 95% CI 0.18 to 0.37, *p*<0.001) but not in studies that trained their participants more than three times per week (*g* = 0.07, 95% CI −0.06 to 0.19, *p* = 0.28; *Q* = 9.082, df = 2, *p* = 0.011). Within-subgroup heterogeneity was low for training either once per week (*I* ^2^ = 0%, 95% CI 0% to 0%; *Q* = 1.04, df = 5, *p* = 0.96) or 2–3 times per week (18.93%, 95% CI 0% to 49%; *Q* = 34.54, df = 28, *p* = 0.18). The intersection of these two moderators (group- versus home-based administration and number of sessions per week), i.e., group-based CCT studies that administered 2–3 sessions per week, comprised a subset of *k* = 25 studies and produced a similar effect estimate: g = 0.29, 95% CI 0.18 to 0.39, *p*<0.001; *Q* statistic for within-subgroup heterogeneity = 30.84, df = 24, *p* = 0.16; *I* ^2^ = 22.18%, CI 0% to 52.44%.

**Figure 12:**
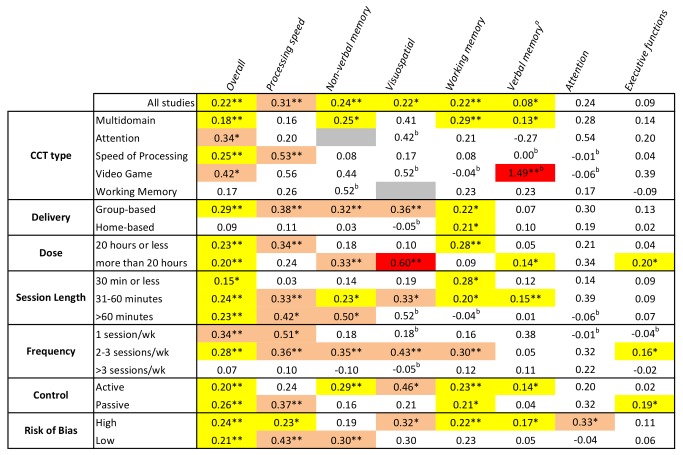
Overview of efficacy and moderators of efficacy for CCT in older adults. Numbers refer to SMDs from an individual meta-analysis (see Figures S2, S3, S4, S5, S6, S7, S8 for details). Colored cells indicate significant outcomes, with effect sizes color coded: yellow, *g*<0.3; pink, *g* = 0.3–0.6; red, *g*≥0.6. White depicts non-significant results, and grey shows where no studies were available for analysis. *p<0.05, **p<0.01 for within-subgroup results (between-subgroup results are reported in Figures 11 and S2, S3, S4, S5, S6, S7, S8). ^a^Based on a fixed-effects model because of evidence of potential publication bias in these outcomes. ^b^SMD based on a single trial.

A similar sequence of moderator analyses for each cognitive domain can be found in Figures S2, S3, S4, S5, S6, S7, S8. A summary of these outcomes is visually presented in Figure 12, a matrix that shows color-coded SMDs for each cognitive domain by each moderating factor. From this figure it is evident that there is no positive evidence for the efficacy of training involving WM (based on either all studies or by subgroup), nor for training administered more than three sessions per week, for any of the cognitive outcomes in this review. At the domain-specific level, evidence for the efficacy of CCT training at home, training only once per week, or in sessions shorter than 30 min is weak.

## Discussion

CCT research involving healthy older participants has now matured to a substantial literature, encompassing 51 RCTs of reasonable quality. When examined en masse, CCT is effective at enhancing cognitive function in healthy older adults, but small effect sizes are to be expected. By definition this result pertains to the theoretical “average” older person—it is currently not possible to predict whether a given individual's cognitive abilities will improve beyond normal practice effects. More importantly, the efficacy of CCT depends on particular design choices as well as the cognitive outcome of interest. Moderator analyses revealed the inefficacy of home-based training compared to group-based training, as well as training more than three times a week. Domain-specific analyses found evidence of efficacy for nonverbal memory, processing speed, WM, and visuospatial outcomes, but not for attention and executive functions. Equally important, we found consistent evidence for the likely inefficacy of WM training and the use of brief training sessions.

Evidence of possible publication bias was found only for reports of verbal memory outcomes. In this case a more conservative fixed-effects model was used and found that CCT efficacy in this domain is weak at best (*g* = 0.08, 95% CI 0.01 to 0.15). Somewhat atypically, the funnel plot for SOP outcomes found that the largest trials tended to find the largest effect sizes. Given that more than half of all participants in this systematic review undertook speed-based training [47],[50]–[55],[59],[69], whose efficacy does not generalize beyond speed-based outcomes (Figure 12), it is possible this is a peculiarity of studies focused on speed training and testing.

Analyses of verbal memory and executive outcomes were sufficiently powered, encompassing 23 and 29 trials, respectively, yet yielded negligible effects. Whilst we recognize that no universal consensus is possible when classifying cognitive tests to particular domains, we consulted a widely cited textbook [21] for this task (see Table S1), and so the negative results for verbal memory and executive outcomes likely represent deficits in the efficacy of CCT in healthy older individuals. Further research aimed at assessing the therapeutic responsiveness of these two key cognitive domains is required, along with development of new and better targeted CCT technology. Consideration should also be given to combining CCT with other effective interventions, such as physical exercise for executive functions [89] and memory strategy training for verbal memory [90].

At the same time, the therapeutic value of several commonly implemented CCT design choices come under question. We found that WM training alone was not effective in healthy older adults, similar to the limited effects reported in a recent meta-analysis in children and young adults [91]. The Finnish Geriatric Intervention Study to Prevent Cognitive Impairment and Disability (FINGER) [92] is a major trial in progress that involves WM training along with other lifestyle-based interventions, and may shed light on the utility (or lack thereof) of this kind of CCT.

One of the attractions of home-based (often Internet-delivered) CCT is the ability to administer a customized and adaptive intervention in the individual's home, with potential for decreased implementation cost [9] and the facility to target the frail and immobile. However, our formal moderator analysis (based on the conservative *Q* statistic) revealed a significant interaction between delivery setting and therapeutic outcome, whereby group-based delivery was effective (*g* = 0.29, 95% CI 0.21 to 0.38) and home-based delivery was not (g = 0.09, 95% CI −0.02 to 0.21). A high degree of consistency amongst group-based training studies suggests that this conclusion is robust (Figure 11). If translated to Mini-Mental State Examination scores, this group-based CCT effect may approximate an average relative improvement of one point [93]. Potentially relevant practice variables when conducting group-based CCT include direct supervision by a trainer to help ensure adherence, treatment fidelity, and compliance; provision of motivational support and encouragement to master challenging tasks that are otherwise easy to avoid; problem solving of IT issues; and nonspecific factors such as social interaction. Indeed, a meta-analysis of memory training in older adults also found that group-based administration was a moderating factor [94]. When conducting CCT, group setting may therefore represent a key therapeutic consideration. Conversely, the popular model of purely home-based training is unlikely to result in cognitive benefits in unimpaired older adults. Future studies may wish to investigate the value of combining initial group-based administration with more long-lasting home-based CCT, as well as test emerging technologies that allow remote clinical supervision and interaction via social media.

We also found interesting evidence for the importance of correct CCT dose. The results suggested that short sessions of less than 30 min may be ineffective, possibly because synaptic plasticity is more likely after 30–60 min of stimulation [95]. By contrast, our analysis clearly identified that training more than three times per week neutralizes CCT efficacy (Figure 11). It is possible that there is a maximal dose for CCT, after which factors such as cognitive fatigue [96] may interfere with training gains. This might not be unique to older persons, as comparative studies in children [97] and young adults [98] have linked spaced training schedules with greater CCT efficacy.

### Limitations

To our knowledge, this is the first quantitative meta-analysis of RCTs in the defined field of CCT in cognitively healthy older adults. As opposed to previous reviews that included various cognitive interventions and research designs [9],[14]–[18], we employed strict eligibility criteria, allowing comparison of results across cognitive domains as well as testing of the impact of design factors. However, by way of limitation our results do not necessarily generalize to older impaired persons, especially the high-risk MCI population, where results appear to be mixed [99],[100]. This review also focused on change in neuropsychological measures immediately after the end of training; it therefore provides no indication about the durability of the observed gains, nor their transfer into real-life outcomes such as independence, quality of life, daily functioning, or risk of long-term cognitive morbidity. Because individual RCTs typically report multiple cognitive test results for a particular cognitive domain, these were combined statistically (as per prior practice [30],[31]), but this approach is blind to the relative psychometric merits of the individual tests. More sophisticated analyses may therefore need to be developed that incorporate test-specific weightings when combining test outcomes. Finally, whilst the CCT literature is now substantive in terms of the number of RCTs (*k* = 51), the typical trial was modest in size (median *N* = 45). Future studies incorporating supervised group-based delivery *and* a session frequency of 2–3 sessions per week can anticipate an approximate effect size of *g* = 0.29, suggesting that a sample of 87 is sufficient to designate power at 0.8 and allow for 15% attrition.

### Conclusions

Discussion of CCT tends to focus on whether it “works” rather than on what factors may contribute to efficacy and inefficacy [13],[101]. This systematic review indicates that its overall effect on cognitive performance in healthy older adults is positive but small, and it is ineffective for executive functions and verbal memory. Accurate individual predictions are not possible. More importantly, our analysis shows that efficacy varies by cognitive outcome and is to a large extent determined by design choices. In general, group-based CCT is effective but home-based CCT is not, and training more than three times a week is counterproductive. Consistently ineffective design choices should therefore be avoided. Improving executive functions or verbal memory may require development of new technology or combined interventions. There remains great scope for additional research to further enhance this non-pharmacological intervention for older individuals.

## Supporting Information

Figure S1 **Funnel plots.** (A) Verbal memory, (B) nonverbal memory, (C) WM, (D) processing speed, (E) executive functions, (F) attention, and (G) visuospatial skills.
(TIF)

Figure S2 **Moderators of efficacy of CCT for verbal memory.** ^a^ *Q-*test for between-group heterogeneity, fixed-effects model. ^b^Total number of training hours.
(TIF)

Figure S3 **Moderators of efficacy of CCT for nonverbal memory.** ^a^ *Q-*test for between-group heterogeneity, mixed-effects model. ^b^Total number of training hours.
(TIF)

Figure S4 **Moderators of efficacy of CCT for working memory.** ^a^ *Q-*test for between-group heterogeneity, mixed-effects model. ^b^One study that combined data from both home- and group-based training [55] was excluded from this analysis. ^c^Total number of training hours.
(TIF)

Figure S5 **Moderators of efficacy of CCT for processing speed.** ^a^ *Q-*test for between-group heterogeneity, mixed-effects model. ^b^One study that combined data from both home- and group-based training [55] was excluded from this analysis. ^c^Total number of training hours. ^d^Session length could not be determined for one study [48].
(TIF)

Figure S6 **Moderators of efficacy of CCT for executive function.** ^a^ *Q-*test for between-group heterogeneity, mixed-effects model. ^b^Total number of training hours. ^c^Session length could not be determined for one study [48].
(TIF)

Figure S7 **Moderators of efficacy of CCT for attention.** ^a^ *Q-*test for between-group heterogeneity, mixed-effects model. ^b^Total number of training hours.
(TIF)

Figure S8 **Moderators of efficacy of CCT for visuospatial skills.** ^a^ *Q-*test for between-group heterogeneity, mixed-effects model. ^b^Total number of training hours.
(TIF)

Table S1 **Classification of neuropsychological outcomes.**
(DOCX)

Table S2 **Group data extraction and results of individual studies.**
(DOCX)

Table S3 **Data provided by primary authors.**
(DOCX)

Table S4 **Risk of bias within studies.**
(DOCX)

Table S5 **Results of sensitivity analyses.**
(DOCX)

Checklist S1 **PRISMA checklist.**
(DOC)

Dataset S1 **Raw effect size and moderator data for overall (combined) and domain-specific results.**
(XLSX)

Protocol S1 **Study protocol.**
(DOCX)

## Abbreviations

CCT: computerized cognitive training
CMA: Comprehensive Meta-Analysis
MCI: mild cognitive impairment
PEDro: Physiotherapy Evidence Database
RCT: randomized controlled trial
SD: standard deviation
SMD: standardized mean difference
SOP: speed of processing
WM: working memory
