# Supplementary figures and images for: Computerized Cognitive Training in Cognitively Healthy Older Adults: A Systematic Review and Meta-Analysis of Effect Modifiers
Source: PLoS Med. 2014 Nov 18;11(11):e1001756. doi: 10.1371/journal.pmed.1001756 (PMC4236015; doi:10.1371/journal.pmed.1001756)

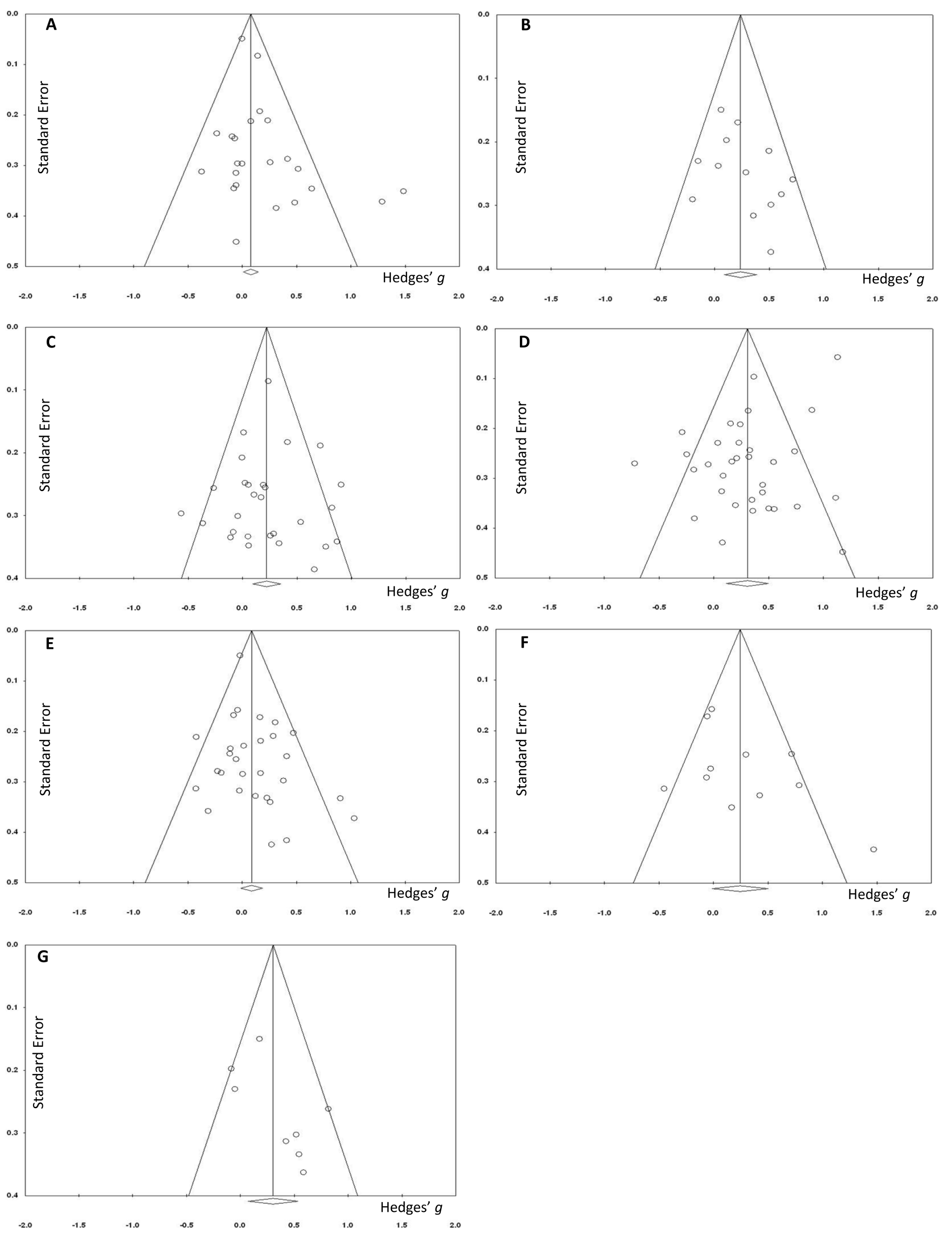

Supplement: Figure S1 — Funnel plots. (A) Verbal memory, (B) nonverbal memory, (C) WM, (D) processing speed, (E) executive functions, (F) attention, and (G) visuospatial skills. (TIF) [file pmed.1001756.s001.tif]

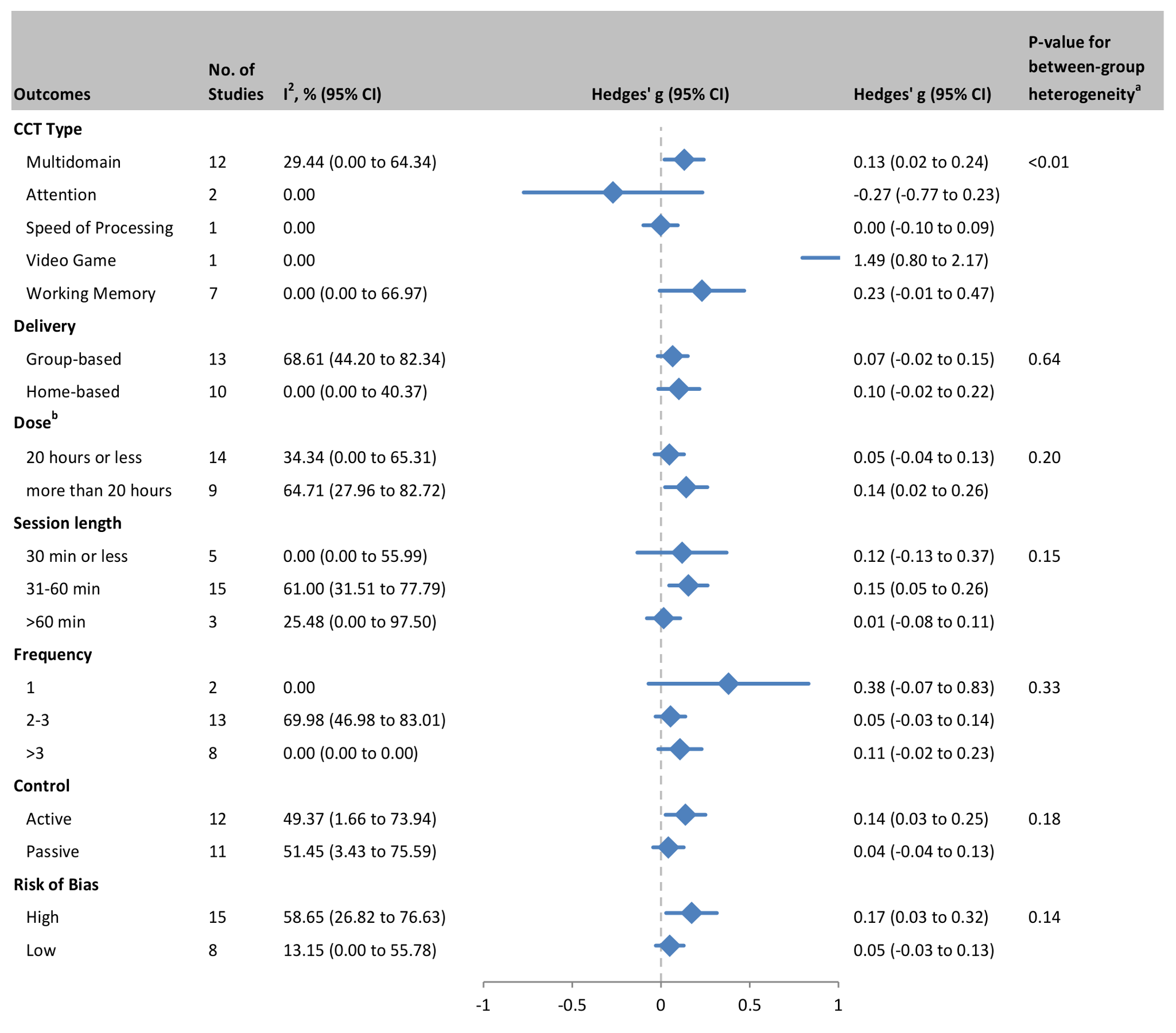

Supplement: Figure S2 — Moderators of efficacy of CCT for verbal memory. a Q-test for between-group heterogeneity, fixed-effects model. bTotal number of training hours. (TIF) [file pmed.1001756.s002.tif]

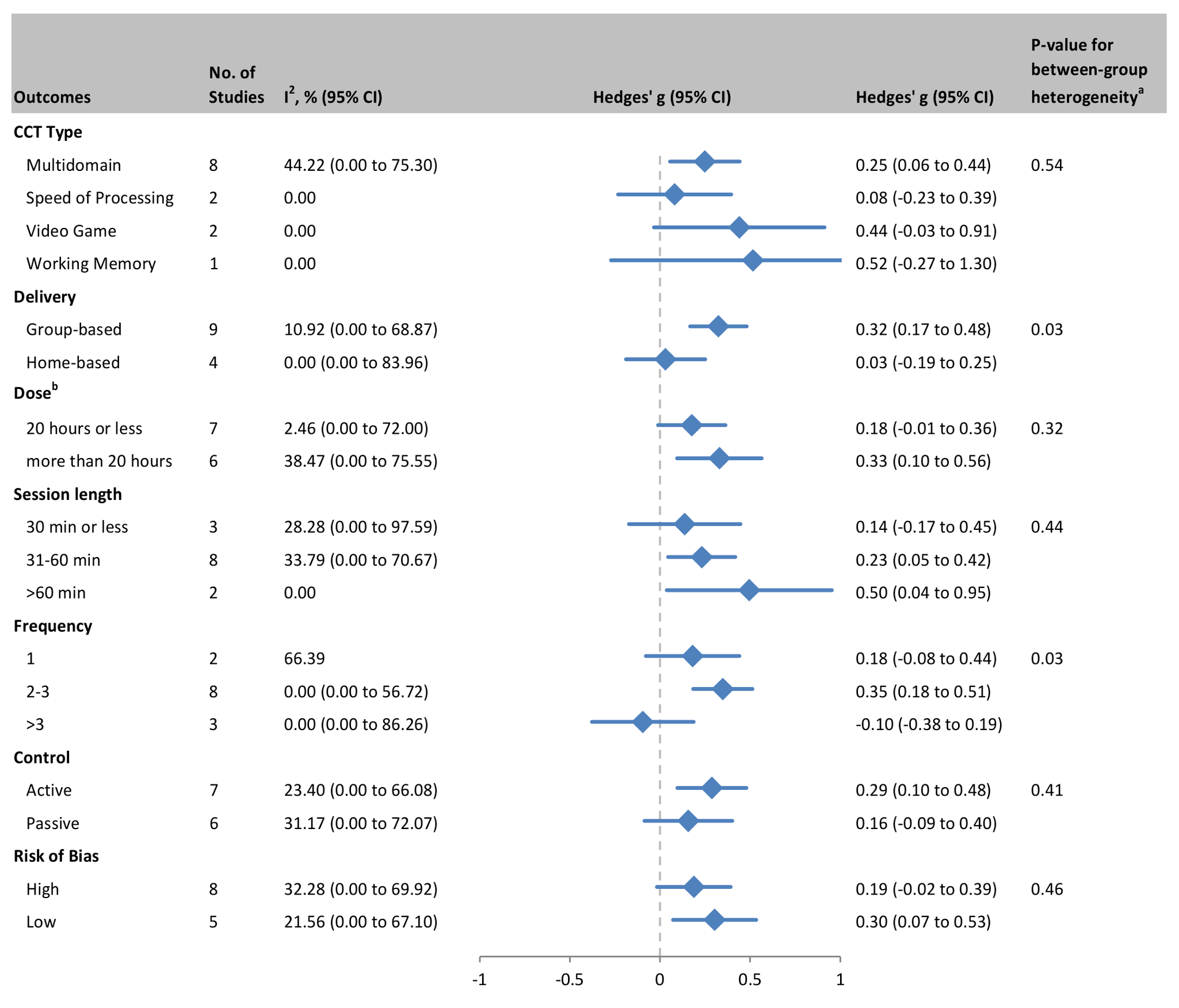

Supplement: Figure S3 — Moderators of efficacy of CCT for nonverbal memory. a Q-test for between-group heterogeneity, mixed-effects model. bTotal number of training hours. (TIF) [file pmed.1001756.s003.tif]

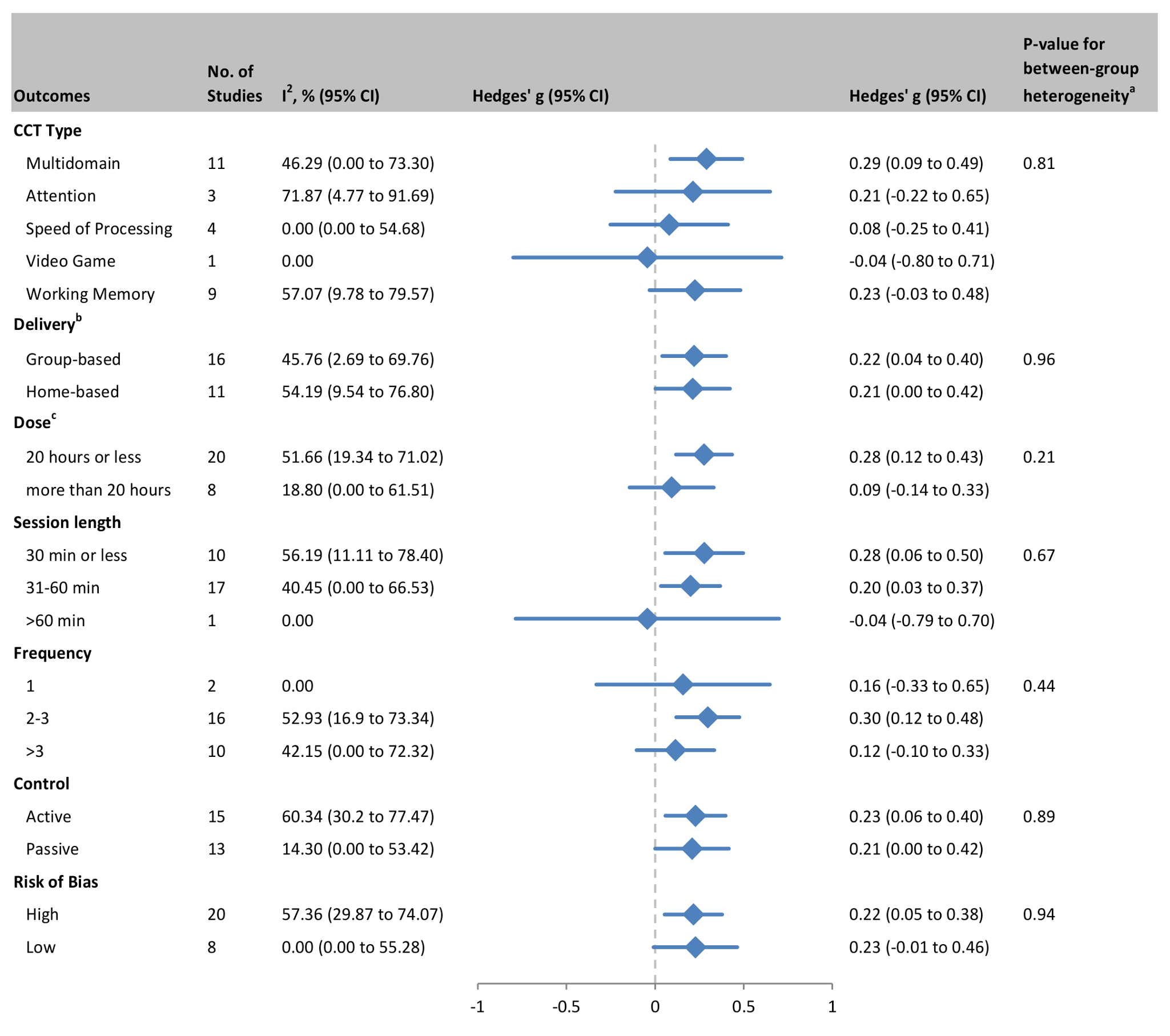

Supplement: Figure S4 — Moderators of efficacy of CCT for working memory. a Q-test for between-group heterogeneity, mixed-effects model. bOne study that combined data from both home- and group-based training [55] was excluded from this analysis. cTotal number of training hours. (TIF) [file pmed.1001756.s004.tif]

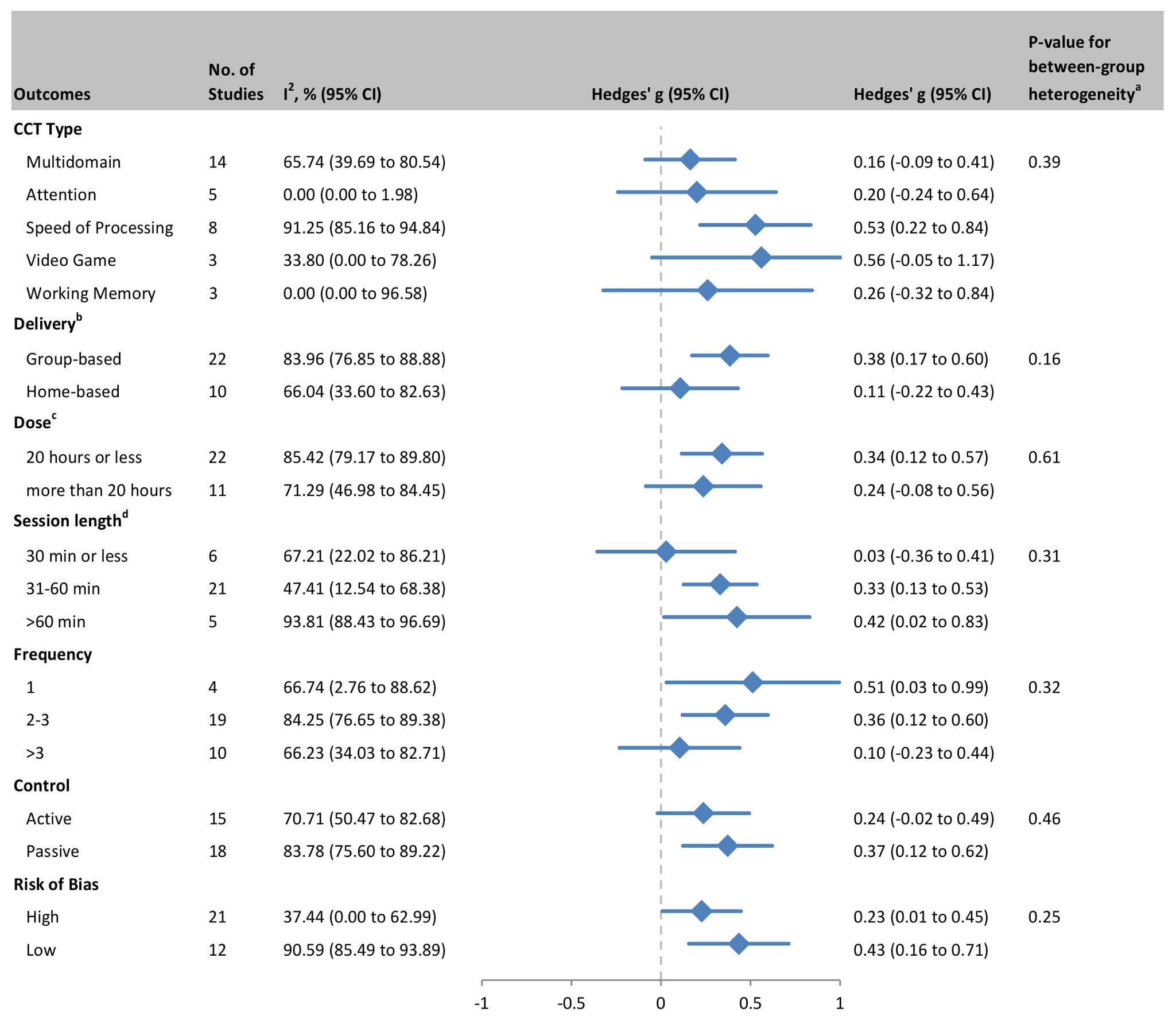

Supplement: Figure S5 — Moderators of efficacy of CCT for processing speed. a Q-test for between-group heterogeneity, mixed-effects model. bOne study that combined data from both home- and group-based training [55] was excluded from this analysis. cTotal number of training hours. dSession length could not be determined for one study [48]. (TIF) [file pmed.1001756.s005.tif]

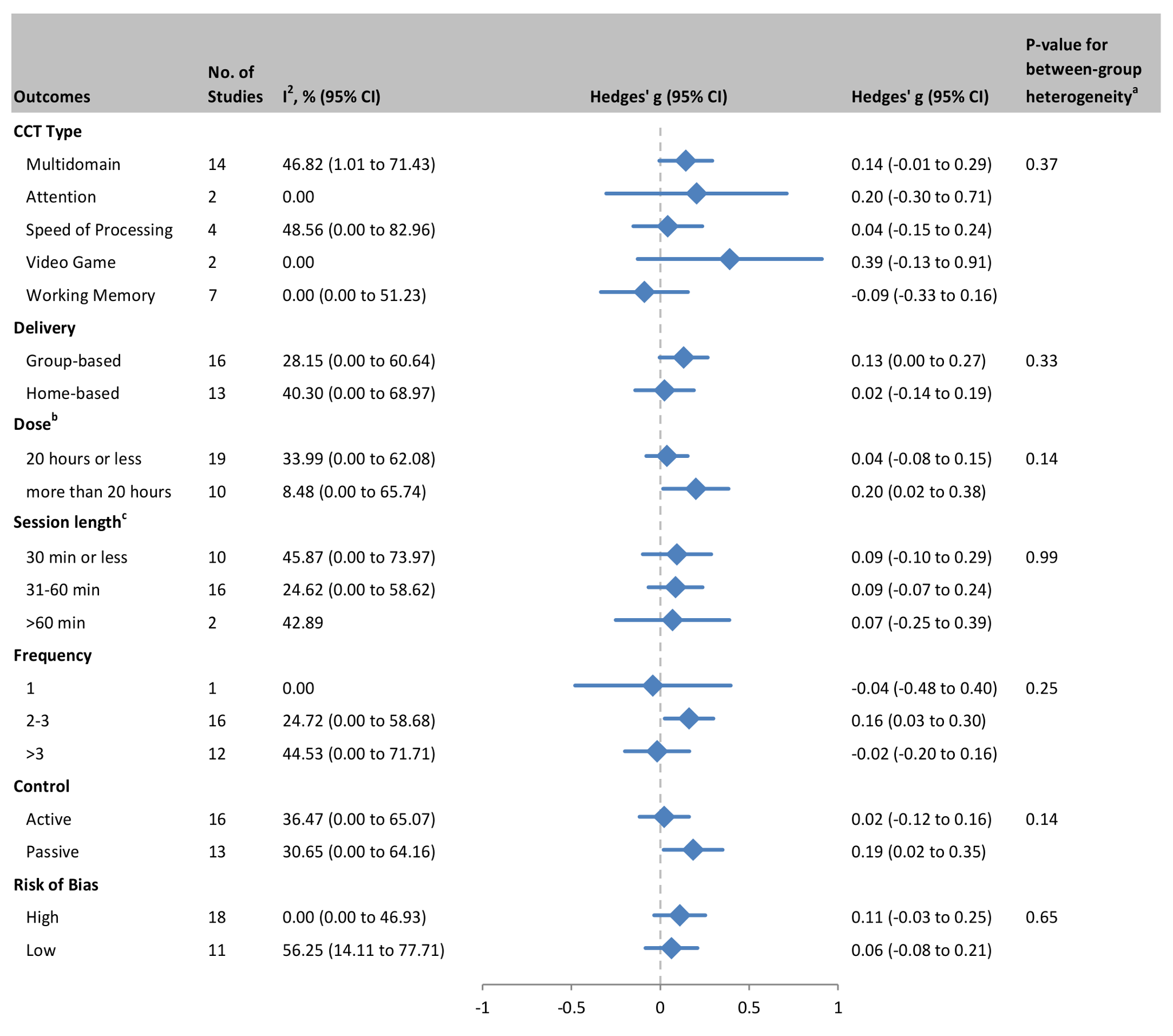

Supplement: Figure S6 — Moderators of efficacy of CCT for executive function. a Q-test for between-group heterogeneity, mixed-effects model. bTotal number of training hours. cSession length could not be determined for one study [48]. (TIF) [file pmed.1001756.s006.tif]

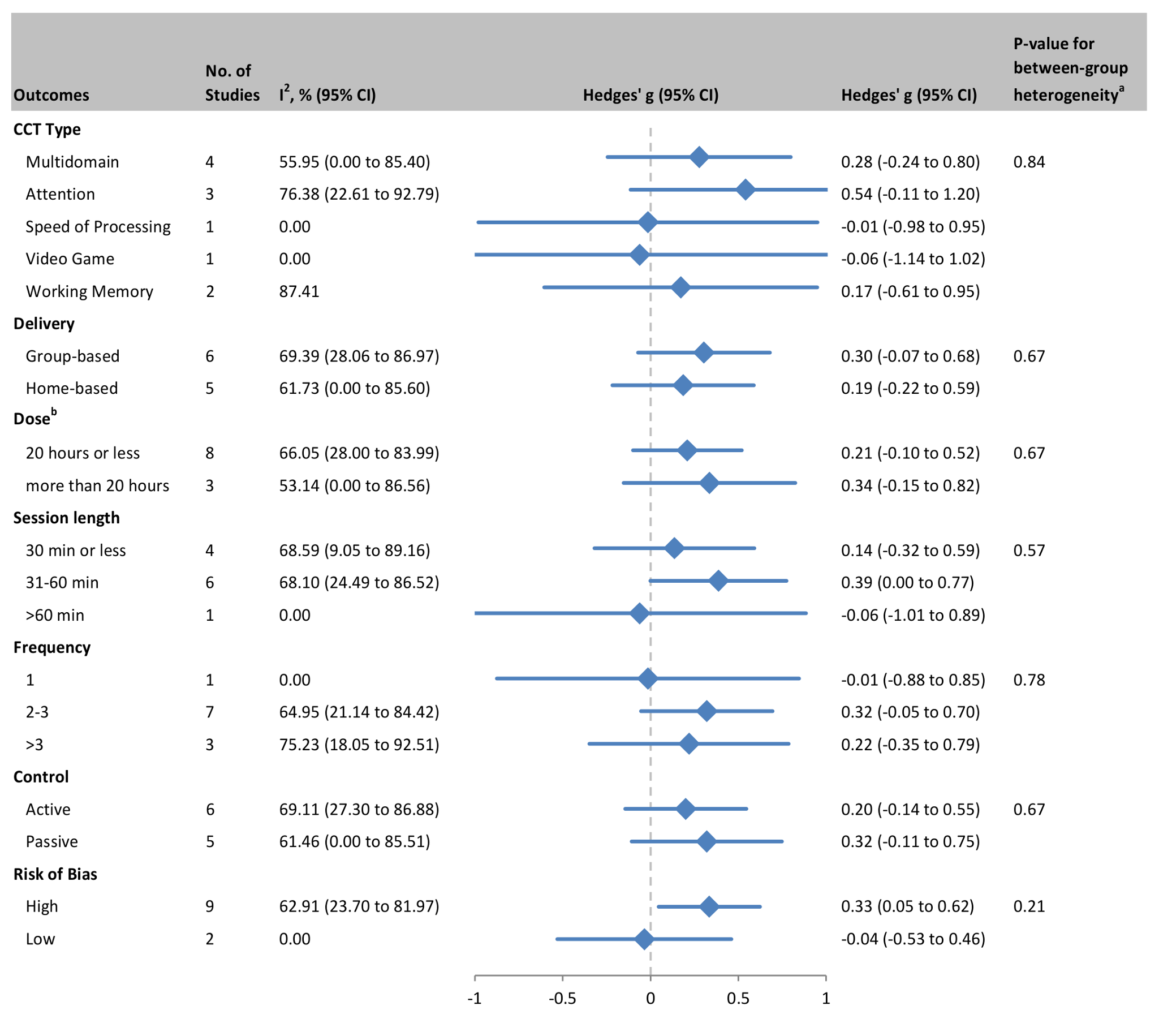

Supplement: Figure S7 — Moderators of efficacy of CCT for attention. a Q-test for between-group heterogeneity, mixed-effects model. bTotal number of training hours. (TIF) [file pmed.1001756.s007.tif]

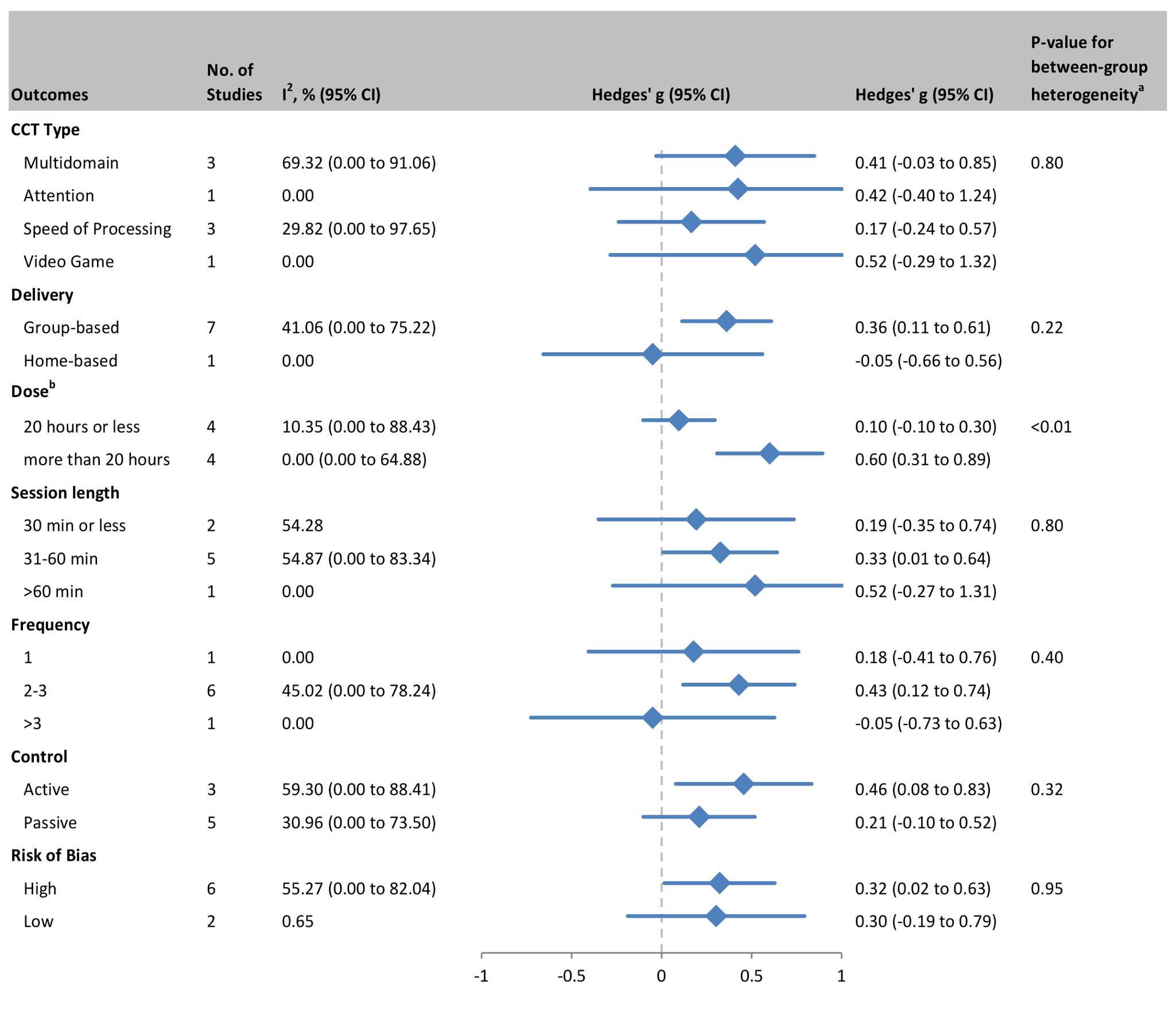

Supplement: Figure S8 — Moderators of efficacy of CCT for visuospatial skills. a Q-test for between-group heterogeneity, mixed-effects model. bTotal number of training hours. (TIF) [file pmed.1001756.s008.tif]
