# Supplementary material for: Computerized Cognitive Training in Cognitively Healthy Older Adults: A Systematic Review and Meta-Analysis of Effect Modifiers
Source: PLoS Med. 2014 Nov 18;11(11):e1001756. doi: 10.1371/journal.pmed.1001756 (PMC4236015; doi:10.1371/journal.pmed.1001756)
Supplement: Table S1 — Classification of neuropsychological outcomes. (DOCX) [file pmed.1001756.s009.docx]

| **Table S1: Classification of neuropsychological outcomes** | | |  |
| --- | --- | --- | --- |
| **Attention** |  |  |  |
| Attentional Blink | **Executive function (cont)** | **Executive function (cont)** | **Non-verbal Memory (cont)** |
| Avoiding Distracters | Executive Functions | Trail Making Test (B, B-A) | Visual Free Recall |
| Change detection task | Figural Analogies | Word Series Correct | Visual STM |
| Divided Attention | Figural Relations |  | Visuospatial Learning |
| Dual Task | Figure Analysis | **Global Cognition** |  |
| Enumeration | Frontal Assessment Battery | Mini Mental State Examination | **Speed** |
| Filter task | Inhibition |  | Cancellation |
| Focused Attention | Kinship Integration | **Language** | Complex Accuracy |
| Functional FOV | Letter Fluency | Boston Naming Test | Complex Choice Reaction |
| Global Local | Letter Series | Naming | Complex RT |
| Oddball | Letter Sets |  | Decision time: choice RT |
| PASAT | Matrix Reasoning Test | **Non-verbal Memory** | Digit cancellation task |
| Selective Attention | Mental Flexibility | Benton Visual Retention | Digit Comparison |
| Smiling Faces | Number Series | Continuous Paired Associations | Digit Symbol |
| Sustained Attention | Planning | Corsi Block Tapping | Digit Symbol Coding (WAIS-III) |
| Test of Attentional Performance | Plus-Minus | Mindstreams Nonverbal Memory | Digit Symbol Substitution |
| Test of Everyday Attention | Raven's Progressive Matrices | Faces I&II (WMS) | Factors of 7 |
| TOVA RT variability change | Self-Oredered Pointing Task | Face-name learning | Field of Vision |
|  | Set-Switch Task | Family Pictures I&II (WMS) | Finding A's |
| **Executive function** | Shifting | Global Visual Memory | Identical Pictures |
| Alternating Runs | Stockings of Cambridge | Memory Recall | Identification |
| Card Prediction | Stop-signal | Memory Recognition | Mindstreams Information Processing Speed |
| Category & Verbal Fluency | Stopping | One Card Learning |  |
| COWAT | Stroop | Place-word learning | Letter Comparison |
| Eriksen Flanker Test | Task Switching | Recognition of figures-list | Letter/Pattern Comparison |
| Everyday Cognitive Battery Reasoning | Test of Non-Verbal Intelligence | Rey Figure (Recall) | Monitoring |
|  |  |  |  |
|  |  |  |  |
| **Speed (cont)** | **Verbal Memory** | **Verbal Memory (cont)** | **Working memory (cont)** |
| Movement time: choice RT | 16 Word Recall | Verbal Paired Recognition (WMS) | Digit Span Forwards (Wechsler) |
| Name Comparison | CERAD | Verbal Pairs Total (WMS-III) | Letter Number Sequence (Wechsler) |
| Naming Symbols | CVLT | W-J III Auditory ST memory | Mental Counters |
| Number Comparison | Delayed Memory (Buschke-Fuld) | Word Recall | N-Back Task |
| Pattern Comparison | Mindstreams Verbal Memory |  | Operation span |
| Perceptual Discrimination | Delayed Verbal Pairs (WMS-III) | **Visuospatial** | Reading Span |
| Perceptual Speed | Everyday Cognitive Battery Recognition | Block Design (Wechsler) | Span Board Backward (WAIS-R) |
| Reaction time | HVLT | Direction Headings | Spatial N-Back |
| Road Sign Test | Immediate Memory (Buschke-Fuld) | Facial Recognition | Spatial Span (WMS) |
| Simple Accuracy | Letter Memory | Hand-eye Coordination | Spatial WM (SUCCAB) |
| Simple Reaction Time | Logical Memory I&II (WMS-III) | Mental Rotation | Updating |
| Simple Choice Reaction | Logical Memory Recognition (WMS) | Motor-free Visual Perception Test | Verbal Complex Span |
| Speed of visual-spatial IP | Meaningful Memory | Rey Figure (Copy) | Visuospatial Working Memory |
| Stimulus Detection | Multiple Measures Memory Test |  | WM Task |
| Symbol Digit Modalities Test | Number Memory | **Working memory** |  |
| Symbol Search (WAIS-III) | One Word Learning | Arithmetic (WAIS-III) |  |
| Tapping | Paired associations | Auditory Working Memory |  |
| TOVA- RT | Paired-associates recall | Backwards spatial span test |  |
| Trails A | RAVLT | Binding |  |
| UFOV | RBANS | Block Span |  |
| Visual Scanning | RBMT | Computation span |  |
| Visual Search Task | Recall of concrete nouns | Counting Span |  |
| WJ-III Visual Matching | Recognition of word-list | Delayed-recognition WM task |  |
|  | Story recall | Digit Span Backwards (Wechsler) |  |
|  | Verbal Free Recall |  |  |
|  | Verbal Paired Associated I&II (WMS) |  |  |
