# Supplementary material for: Computerized Cognitive Training in Cognitively Healthy Older Adults: A Systematic Review and Meta-Analysis of Effect Modifiers
Source: PLoS Med. 2014 Nov 18;11(11):e1001756. doi: 10.1371/journal.pmed.1001756 (PMC4236015; doi:10.1371/journal.pmed.1001756)
Supplement: Table S2 — Group data extraction and results of individual studies. (DOCX) [file pmed.1001756.s010.docx]

**Table S2: Group data extraction and results of individual studies**

| Study | Original group labels | Groups included in analysis | Comments | Results (Hedges’ *g*) |
| --- | --- | --- | --- | --- |
| Ackerman 2010[1] | 1. Wii first 2. Reading first | CCT – group 1  Control – group 2 | Crossover trial comparing CCT to reading practice. Only data from the first phase (‘Interim Test’, i.e., after half of the participants completed CCT and the other half completed reading) were extracted. | Overall: -0.35 (-0.73 to 0.04)  Executive Functions: -0.42 (-0.84 to -0.01)  Processing Speed: -0.29 (-0.70 to 0.12) |
| Anderson 2013[2] | 1. Auditory training 2. Active control | CCT – group 1  Control – group 2 | The control group watched educational DVDs and answered questions about their content. | Overall: 3.90 (3.15 to 4.65)  Verbal Memory: 3.82 (3.02 to 4.62)  Processing speed: 3.98 (3.16 to 4.81) |
| Anguera 2013[3] | 1. Multitasking training 2. Single task training 3. No-contact control | CCT – group 1  Control – group 2 | Passive control condition. | Overall: 0.55 (-0.05 to 1.15)  Attention: 0.43 (-0.22 to 1.07)  Processing Speed: 0.45 (-0.20 to 1.09)  Working Memory: 0.87 (0.20 to 1.54) |
| Ball 2002[4] | 1. Memory training 2. Reasoning training 3. Speed training 4. Control | CCT – group 3  Control – group 4 | Passive control condition. Groups 1 and 2 did not receive computerised training. | Overall: 0.13 (0.04 to 0.22)  Executive Functions: -0.02 (-0.12 to 0.08)  Processing Speed: 1.13 (1.02 to 1.24)  Verbal Memory: 0.00 (-0.10 to 0.09) |
| Barnes 2013[5] | 1. MA-C – EX-C 2. MA-C – EX-I 3. MA-I – EX-C 4. MA-I – EX-I | CCT – group 3  Control – group 1 | MA=Mental activity; EX=Exercise; I=intervention, C=control. 2x2 design. Groups 2 and 4 were excluded from the analysis in order to minimise and possible effect of the physical exercise intervention. The MA-I – EX-C and MA-C – EX-C groups received low-intensity physical training (stretching, toning, strength). Participants in the MA-C group watched educational DVDs and answered questions about them. | Overall: -0.02 (-0.43 to 0.40)  Executive Functions: 0.01 (-0.43 to 0.46)  Processing Speed: 0.04 (-0.41 to 0.49)  Verbal Memory: -0.23 (-0.70 to 0.23) |
| Basak 2008[6] | 1. Rise of Nations 2. Control | CCT – group 1  Control – group 2 | Passive control condition. | Attention: -0.06 (-0.64 to 0.51)  Overall: 0.20 (-0.33 to 0.74)  Executive Functions: 0.38 (-0.20 to 0.96)  Non-Verbal Memory: 0.36 (-0.26 to 0.98)  Visuospatial Skills: 0.52 (-0.07 to 1.11)  Working Memory: -0.04 (-0.63 to 0.55) |
| Belchior 2013[7] | 1. Medal of Honor 2. SOP training 3. Tetris 4. No-contact control | Study 1:  CCT – group 1  Control – group 4  Study 2:  CCT – group 2  Control – group 3 | SOP=Speed of Processing | Overall: 0.20 (-0.47 to 0.87)  Processing Speed: 0.20 (-0.50 to 0.89)  Overall: 0.07 (-0.54 to 0.69)  Processing Speed: 0.07 (-0.57 to 0.71) |
| Berry 2010[8] | 1. Training 2. Control | CCT – group 1  Control – group 2 | Passive control condition. | Overall: 0.39 (-0.24 to 1.03)  Processing Speed: 0.50 (-0.21 to 1.21)  Working Memory: 0.34 (-0.34 to 1.01) |
| Boot 2013[9] | 1. Control 2. Brain fitness 3. Action game | CCT – group 2  Control – group 1 | The action game group was omitted from the analysis due to lack of additional control group | Overall: -0.09 (-0.61 to 0.43)  Executive Functions: 0.00 (-0.55 to 0.56)  Non-Verbal Memory: -0.20 (-0.77 to 0.37)  Processing Speed: -0.18 (-0.74 to 0.37)  Verbal Memory: 0.00 (-0.58 to 0.58) |
| Bottiroli 2009[10] | 1. Training 2. Waiting-list | CCT – group 1  Control – group 2 | Passive control condition. | Overall: 0.53 (0.02 to 1.05)  Non-Verbal Memory: 0.61 (0.06 to 1.17)  Verbal Memory: 0.42 (-0.15 to 0.98) |
| Bozoki 2013[11] | 1. Active 2. Control | CCT – group 1  Control – group 2 | Participants in the active control group received educational materials. | Overall: 0.03 (-0.40 to 0.46)  Executive Functions: -0.06 (-0.56 to 0.45)  Non-Verbal Memory: 0.03 (-0.43 to 0.50)  Processing Speed: 0.33 (-0.15 to 0.81)  Verbal Memory: -0.09 (-0.57 to 0.38)  Working Memory: -0.26 (-0.77 to 0.24) |
| Brehmer 2012[12] | 1. Adaptive training younger adults 2. Adaptive training older adults 3. Low-level practice younger adults 4. Low-level practice older adults | CCT – group 2  Control – group 4 | Participants in the active control group received low-level non-adaptive version of the WM training program. | Overall: 0.46 (-0.06 to 0.97)  Attention: 0.79 (0.18 to 1.39)  Executive Functions: -0.19 (-0.75 to 0.36)  Verbal Memory: -0.04 (-0.63 to 0.54)  Working Memory: 0.82 (0.26 to 1.39) |
| Burki 2014[13] | 1. Younger adults WM 2. Younger adults implicit 3. Younger adults no-contact 4. Older adults WM 5. Older adults implicit 6. Older adults no-contact | CCT - group 4  Control - group 6 | The implicit sequence learning training group was excluded from the analysis due to lack of additional control group. | Overall: 0.04 (-0.46 to 0.53)  Executive Functions: -0.23 (-0.77 to 0.32)  Processing Speed: -0.05 (-0.58 to 0.49)  Working Memory: 0.17 (-0.36 to 0.7) |
| Buschkuehl 2008[14] | 1. Experimental 2. Control | CCT – group 1  Control – group 2 | Participants in the control group received eccentric muscle training. | Overall: 0.51 (0.07 to 0.95)  Non-Verbal Memory: 0.29 (-0.20 to 0.77)  Verbal Memory: -0.07 (-0.55 to 0.42)  Working Memory: 0.91 (0.41 to 1.40) |
| Casutt 2014[15] | 1. Simulator training 2. Cognitive training 3. No training | CCT - group 2  Control - group 3 | Participants in the simulator training group received driving simulator training. | Overall: 0.11 (-0.38 to 0.60)  Attention: -0.02 (-0.56 to 0.51)  Processing Speed: 0.17 (-0.36 to 0.69) |
| Colzato 2011[16] | 1. Met/- experimental 2. Met/- control 3. Val/Val experimental 4. Val/Val control | CCT – groups 1+3  Control – group 2+4 | Results of groups 1+3 and 2+4 were combined using the formulae suggested by the Cochrane Collaboration^15^. Participants in the control group watched documentaries and answered questions about them. | Overall: -0.72 (-1.24 to -0.21)  Processing Speed: -0.72 (-1.25 to -0.19) |
| Dahlin 2008[17] | 1. Young trained 2. Young control 3. Old trained 4. Old control | CCT – group 3  Control – group 4 | Passive control condition. | Overall: 0.05 (-0.56 to 0.66)  Executive Functions: 0.26 (-0.41 to 0.93)  Processing Speed: 0.36 (-0.36 to 1.07)  Verbal Memory: -0.08 (-0.75 to 0.60)  Working Memory: -0.11 (-0.77 to 0.55) |
| Dustman 1992[18] | 1. Control 2. Movie 3. Videogame | CCT – group 3  Control – group 1 | Participants in the movie group attended group movie viewing sessions. | Overall: 0.61 (0.07 to 1.15)  Non-Verbal Memory: 0.52 (-0.07 to 1.10)  Processing Speed: 0.45 (-0.17 to 1.06)  Verbal Memory: 1.49 (0.80 to 2.17) |
| Edwards 2002[19] | 1. Control 2. Training | CCT – group 2  Control – group 1 | Passive control condition. | Overall: 0.17 (-0.18 to 0.52)  Executive Functions: 0.47 (0.07 to 0.87)  Non-Verbal Memory: 0.11 (-0.28 to 0.50)  Processing Speed: 0.15 (-0.22 to 0.53)  Visuospatial Skills: -0.08 (-0.47 to 0.30)  Working Memory: 0.00 (-0.41 to 0.40) |
| Edwards 2005[20] | 1. Speed 2. Internet | CCT – group 1  Control – group 2 | Participants in the internet group received computer use training. | Overall: 0.19 (-0.11 to 0.49)  Executive Functions: -0.08 (-0.41 to 0.25)  Processing Speed: 0.31 (-0.01 to 0.64)  Working Memory: 0.01 (-0.32 to 0.34) |
| Edwards 2013[21] | 1. InSight training 2. Controls | CCT – group 1  Control – group 2 | Passive control condition. | Overall: 0.32 (-0.18 to 0.83)  Processing Speed: 0.32 (-0.18 to 0.83) |
| Garcia-Campuzano 2013[22] | 1. Training 2. Control | CCT - group 1  Control - group 2 | Passive control condition. Other cognitive outcomes from this study were under peer-review for publication at the time this review was completed. | Overall: 0.52 (-0.16 to 1.21)  Non-Verbal Memory: 0.52 (-0.22 to 1.25)  Verbal Memory: 0.48 (-0.25 to 1.22)  Working Memory: 0.66 (-0.10 to 1.42) |
| Goldstein 1997[23] | 1. Experimental 2. Control | CCT – group 1  Control – group 2 | Passive control condition. | Overall: 0.79 (0.01 to 1.58)  Executive Functions: 0.41 (-0.41 to 1.23)  Processing Speed: 1.18 (0.30 to 2.06) |
| Heinzel 2013[24] | 1. Young training group 2. Young control group 3. Old training group 4. Old control group | CCT – group 3  Control – group 4 | Passive control condition. | Overall: 0.51 (-0.10 to 1.12)  Executive Functions: 0.23 (-0.42 to 0.88)  Processing Speed: 0.55 (-0.16 to 1.26)  Verbal Memory: 0.64 (-0.04 to 1.32)  Working Memory: 0.77 (0.08 to 1.45) |
| Lampit 2014[25] | 1. CCT 2. Active control | CCT - group 1  Control - group 2 | The study was conducted by the authors of this review | Overall: 0.32 (-0.06 to 0.70)  Executive Functions: 0.29 (-0.12 to 0.70)  Non-Verbal Memory: 0.50 (0.08 to 0.92)  Processing Speed: 0.23 (-0.22 to 0.68)  Verbal Memory: 0.23 (-0.18 to 0.65) |
| Lee 2012[26] | 1. Training 2. Balance exercise | CCT – group 1  Control – group 2 | Participants in the balance exercise group performed movements from a balance evaluation method. | Overall: 0.60 (-0.05 to 1.26)  Visuospatial Skills: 0.59 (-0.13 to 1.30)  Global cognition: 0.62 (-0.09 to 1.33) |
| Legault 2011[27] | 1. Healthy aging 2. Cognitive training 3. Physical activity training 4. Combined intervention | CCT – group 2  Control – group 1 | Participants in the healthy ageing group received health education lectures. | Overall: -0.05 (-0.63 to 0.53)  Executive Functions: -0.02 (-0.65 to 0.60)  Verbal Memory: -0.06 (-0.68 to 0.56)  Working Memory: -0.09 (-0.73 to 0.55) |
| Li 2010[28] | 1. Training 2. Control | CCT – group 1  Control – group 2 | Passive control condition. | Overall: 0.15 (-0.62 to 0.92)  Executive Functions: 0.27 (-0.56 to 1.10)  Processing Speed: 0.08 (-0.76 to 0.92)  Verbal Memory: -0.06 (-0.94 to 0.83) |
| Lussier 2012[29] | 1. Older trained 2. Older control 3. Younger trained 4. Younger control | CCT – group 1  Control – group 2 | Passive control condition. | Overall: 1.47 (0.65 to 2.29)  Attention: 1.47 (0.62 to 2.32) |
| Mahncke 2006[30] | 1. Experimental training 2. Active control 3. No-contact control | CCT – group 1  Control – group 2 | Participants in the active control group watched educational DVDs- | Overall: 0.16 (-0.22 to 0.54)  Verbal Memory: 0.16 (-0.22 to 0.54) |
| Maillot 2012[31] | 1. Control 2. Training | CCT – group 2  Control – group 1 | Passive control condition. | Overall: 0.90 (0.29 to 1.51)  Executive Functions: 0.91 (0.25 to 1.56)  Processing Speed: 1.11 (0.45 to 1.78)  Visuospatial Skills: 0.55 (-0.11 to 1.20)  Working Memory: 0.29 (-0.36 to 0.93) |
| Mayas 2014[32] | 1. Experimental 2. Control | CCT - group 1  Control - group 2 | Social contact control group. | Overall: 0.17 (-0.49 to 0.83)  Attention: 0.17 (-0.52 to 0.86) |
| McAvinue 2013[33] | 1. Trainee 2. Control | CCT – group 1  Control – group 2 | Participants in the active control group received low-level non-adaptive version of the WM training program. | Overall: 0.52 (-0.04 to 1.09)  Verbal Memory: 0.52 (-0.09 to 1.12)  Working Memory: 0.53 (-0.07 to 1.14) |
| Miller 2013[34] | 1. Intervention 2. Control | CCT – group 1  Control – group 2 | Passive control condition. | Overall: 0.07 (-0.31 to 0.46)  Executive Functions: 0.17 (-0.26 to 0.60)  Non-Verbal Memory: -0.15 (-0.60 to 0.30)  Verbal Memory: 0.08 (-0.34 to 0.50)  Visuospatial Skills: -0.05 (-0.50 to 0.40)  Language: 0.21 (-0.25 to 0.66) |
| Nouchi 2012[35] | 1. Brain Age 2. Tetris | CCT – group 1  Control – group 2 | Participants in the Tetris group played the game for the same duration as the Brain Age group. | Overall: 0.57 (-0.07 to 1.20)  Executive Functions: 1.03 (0.30 to 1.76)  Processing Speed: 0.76 (0.06 to 1.46)  Working Memory: 0.06 (-0.63 to 0.74)  Global cognition: 0.05 (-0.67 to 0.77) |
| O’Brien 2013[36] | 1. SOP training 2. No-contact control | CCT – group 1  Control – group 2 | Passive control condition. | Overall: -0.18 (-0.89 to 0.54)  Processing Speed: -0.18 (-0.92 to 0.57) |
| Peng 2012[37] | 1. Control 2. Paper-pencil 3. Computer training | CCT – group 3  Control – group 2 | The paper-pencil group received a paper-based version of the computerised exercises | Overall: 0.33 (-0.16 to 0.81)  Processing Speed: 0.55 (0.02 to 1.07)  Working Memory: 0.11 (-0.42 to 0.63) |
| Peretz 2011[38] | 1. Personalized cognitive training 2. Computer games | CCT – group 1  Control – group 2 | The computer games group played classic computer games for the same duration as the CCT group | Overall: 0.16 (-0.15 to 0.47)  Attention: -0.06 (-0.39 to 0.28)  Executive Functions: 0.16 (-0.17 to 0.50)  Non-Verbal Memory: 0.21 (-0.12 to 0.55)  Working Memory: 0.41 (0.05 to 0.77) |
| Rasmusson 1999[39] | 1. Audiotapes 2. Computer 3. Memory class 4. Wait-list | CCT – group 2  Control – group 4 | Passive control condition. Groups 1 and 2 did not receive computerised training. | Overall: 0.31 (-0.42 to 1.05)  Verbal Memory: 0.31 (-0.44 to 1.07) |
| Richmond 2011[40] | 1. Control 2. Training | CCT – group 2  Control – group 1 | Control participants played online trivia quizzes. | Overall: -0.22 (-0.76 to 0.31)  Attention: -0.45 (-1.07 to 0.16)  Executive Functions: -0.43 (-1.04 to 0.19)  Verbal Memory: 0.26 (-0.32 to 0.83)  Working Memory: -0.56 (-1.15 to 0.02) |
| Sandberg 2014[41] | 1. Training young 2. Control young 3. Training old 4. Control old | CCT - group 3  Control - group 4 | Passive control condition. | Overall: 0.43 (-0.19 to 1.04)  Executive Functions: 0.12 (-0.52 to 0.77)  Verbal Memory: 1.29 (0.56 to 2.02)  Working Memory: 0.26 (-0.40 to 0.91) |
| Shatil 2013[42] | 1. Physical activity 2. Cognitive training 3. Combined training 4. Book reading | CCT – group 2  Control – group 4 | 2x2 design. Groups 1 and 3 were excluded from the analysis in order to minimise and possible effect of the physical exercise intervention. Participants in the book reading group attended book club sessions. | Overall: 0.45 (0.02 to 0.88)  Attention: 0.72 (0.24 to 1.20)  Executive Functions: -0.11 (-0.57 to 0.35)  Non-Verbal Memory: 0.72 (0.21 to 1.23)  Processing Speed: 0.74 (0.26 to 1.22)  Visuospatial Skills: 0.82 (0.30 to 1.33)  Working Memory: 0.05 (-0.44 to 0.55)  Language: 0.77 (0.26 to 1.28) |
| Shatil 2014[43] | 1. Cognitive training 2. Control | CCT - group 1  Control - group 2 | Participants in the control group received a TV-based cognitive stimulation program | Overall: 0.46 (0.12 to 0.79)  Executive Functions: 0.31 (-0.05 to 0.66)  Processing Speed: 0.24 (-0.13 to 0.62)  Working Memory: 0.72 (0.35 to 1.09) |
| Simpson 2012[44] | 1. Brain-training 2. Solitaire | CCT – group 1  Control – group 2 | Participants in the solitaire group played the game on a computer for the same duration as the CCT group. | Overall: 0.11 (-0.50 to 0.72)  Executive Functions: -0.31 (-1.01 to 0.39)  Processing Speed: 0.35 (-0.32 to 1.02)  Verbal Memory: -0.06 (-0.72 to 0.61)  Working Memory: 0.05 (-0.60 to 0.70) |
| Smith 2009[45] | 1. Experimental treatment 2. Active control | CCT – group 1  Control – group 2 | Participants in the active control condition watched educational DVDs and answered questions about them. | Overall: 0.17 (0.02 to 0.32)  Verbal Memory: 0.14 (-0.02 to 0.31)  Working Memory: 0.24 (0.07 to 0.41) |
| Stern 2011[46] | 1. Emphasis change 2. Active control 3. Passive control | CCT – group 2  Control – group 3 | Participants in groups 1 and 2 played Space Fortress. Group 2 was included as the CCT group as this group played the game under standard instructions whereas group 1 played the game under specific experimental conditions (emphasis change), which could have been regarded as attention training. | Overall: 0.10 (-0.42 to 0.62)  Executive Functions: 0.17 (-0.38 to 0.73)  Processing Speed: 0.09 (-0.49 to 0.67)  Verbal Memory: -0.37 (-0.99 to 0.24)  Visuospatial Skills: 0.42 (-0.19 to 1.04)  Working Memory: -0.37 (-0.98 to 0.25) |
| van Muijden 2012[47] | 1. Experimental 2. Documentary | CCT – group 1  Control – group 2 | Participants in the documentary group watched videos and answered question about them. | Overall: 0.20 (-0.24 to 0.65)  Attention: 0.30 (-0.19 to 0.78)  Executive Functions: 0.41 (-0.08 to 0.90)  Processing Speed: -0.25 (-0.74 to 0.25)  Working Memory: 0.19 (-0.30 to 0.68) |
| Vance et al 2007[48] | 1. Social contact control 2. SOP | CCT – group 2  Control – group 1 | Participants in the social contact control group received computer use training. | Overall: 0.28 (0.00 to 0.55)  Attention: -0.01 (-0.32 to 0.29)  Executive Functions: -0.04 (-0.35 to 0.27)  Non-Verbal Memory: 0.06 (-0.23 to 0.35)  Processing Speed: 0.90 (0.58 to 1.22)  Visuospatial Skills: 0.18 (-0.12 to 0.47) |
| von Bastian 2013[49] | 1. Young WM 2. Young AC 3. Old WM 4. Old AC | CCT – group 3  Control – group 4 | The active control group received CCT on domains other than working memory | Overall: -0.05 (-0.49 to 0.40)  Executive Functions: -0.11 (-0.59 to 0.37)  Working Memory: 0.02 (-0.46 to 0.51) |
| Wang 2011[50] | 1. Control 2. Training | CCT – group 2  Control – group 1 | Passive control condition. | Overall: 0.23 (-0.24 to 0.70)  Processing Speed: 0.21 (-0.30 to 0.72)  Working Memory: 0.21 (-0.29 to 0.71)  Global cognition: 0.34 (-0.20 to 0.88) |
| Wolinsky 2011[51] | 1. Road Tour on-site 2. Road Tour on-site with future boosters 3. Attention control on-site 4. Road tour at-home | CCT – groups 1+2  Control – group 3 | Results of groups 1 and 2 were combined using the formulae suggested by the Cochrane Collaboration. Group 4 was excluded from the analysis due to lack of a matching control condition. | Overall: 0.37 (0.18 to 0.56)  Processing Speed: 0.37 (0.18 to 0.56) |

**References**

1. Ackerman PL, Kanfer R, Calderwood C (2010) Use it or lose it? Wii brain exercise practice and reading for domain knowledge. Psychol Aging 25: 753-766.

2. Anderson S, White-Schwoch T, Parbery-Clark A, Kraus N (2013) Reversal of age-related neural timing delays with training. Proc Natl Acad Sci U S A 110: 4357-4362.

3. Anguera JA, Boccanfuso J, Rintoul JL, Al-Hashimi O, Faraji F, et al. (2013) Video game training enhances cognitive control in older adults. Nature 501: 97-101.

4. Ball K, Berch DB, Helmers KF, Jobe JB, Leveck MD, et al. (2002) Effects of cognitive training interventions with older adults: a randomized controlled trial. Jama 288: 2271-2281.

5. Barnes DE, Santos-Modesitt W, Poelke G, Kramer AF, Castro C, et al. (2013) The Mental Activity and eXercise (MAX) trial: a randomized controlled trial to enhance cognitive function in older adults. JAMA Intern Med 173: 797-804.

6. Basak C, Boot WR, Voss MW, Kramer AF (2008) Can training in a real-time strategy video game attenuate cognitive decline in older adults? Psychol Aging 23: 765-777.

7. Belchior P, Marsiske M, Sisco SM, Yam A, Bavelier D, et al. (2013) Video game training to improve selective visual attention in older adults. Comput Human Behav 29: 1318-1324.

8. Berry AS, Zanto TP, Clapp WC, Hardy JL, Delahunt PB, et al. (2010) The influence of perceptual training on working memory in older adults. PLoS One 5: e11537.

9. Boot WR, Champion M, Blakely DP, Wright T, Souders DJ, et al. (2013) Video games as a means to reduce age-related cognitive decline: attitudes, compliance, and effectiveness. Front Psychol 4: 31.

10. Bottiroli S, Cavallini E (2009) Can computer familiarity regulate the benefits of computer-based memory training in normal aging? A study with an Italian sample of older adults. Neuropsychol Dev Cogn B Aging Neuropsychol Cogn 16: 401-418.

11. Bozoki A, Radovanovic M, Winn B, Heeter C, Anthony JC (2013) Effects of a computer-based cognitive exercise program on age-related cognitive decline. Arch Gerontol Geriatr 57: 1-7.

12. Brehmer Y, Westerberg H, Backman L (2012) Working-memory training in younger and older adults: training gains, transfer, and maintenance. Front Hum Neurosci 6: 63.

13. Burki CN, Ludwig C, Chicherio C, de Ribaupierre A (2014) Individual differences in cognitive plasticity: an investigation of training curves in younger and older adults. Psychol Res.

14. Buschkuehl M, Jaeggi SM, Hutchison S, Perrig-Chiello P, Dapp C, et al. (2008) Impact of working memory training on memory performance in old-old adults. Psychol Aging 23: 743-753.

15. Casutt G, Theill N, Martin M, Keller M, Jancke L (2014) The drive-wise project: Driving simulator training increases real driving performance in healthy older drivers. Frontiers in Aging Neuroscience 6.

16. Colzato LS, van Muijden J, Band GP, Hommel B (2011) Genetic Modulation of Training and Transfer in Older Adults: BDNF ValMet Polymorphism is Associated with Wider Useful Field of View. Front Psychol 2: 199.

17. Dahlin E, Nyberg L, Backman L, Neely AS (2008) Plasticity of executive functioning in young and older adults: immediate training gains, transfer, and long-term maintenance. Psychol Aging 23: 720-730.

18. Dustman RE, Emmerson RY, Steinhaus LA, Shearer DE, Dustman TJ (1992) The effects of videogame playing on neuropsychological performance of elderly individuals. J Gerontol 47: P168-171.

19. Edwards JD, Wadley VG, Myers RS, Roenker DL, Cissell GM, et al. (2002) Transfer of a speed of processing intervention to near and far cognitive functions. Gerontology 48: 329-340.

20. Edwards JD, Wadley VG, Vance DE, Wood K, Roenker DL, et al. (2005) The impact of speed of processing training on cognitive and everyday performance. Aging Ment Health 9: 262-271.

21. Edwards JD, Valdes EG, Peronto C, Castora-Binkley M, Alwerdt J, et al. (2013) The Efficacy of InSight Cognitive Training to Improve Useful Field of View Performance: A Brief Report. J Gerontol B Psychol Sci Soc Sci.

22. Garcia-Campuzano MT, Virues-Ortega J, Smith S, Moussavi Z (2013) Effect of cognitive training targeting associative memory in the elderly: A small randomized trial and a longitudinal evaluation. Journal of the American Geriatrics Society 61: 2252-2254.

23. Goldstein J, Cajko L, Oosterbroek M, Michielsen M, Van Houten O, et al. (1997) Video Games and the Elderly. Social Behavior and Personality: an international journal 25: 345-352.

24. Heinzel S, Schulte S, Onken J, Duong QL, Riemer TG, et al. (2014) Working memory training improvements and gains in non-trained cognitive tasks in young and older adults. Neuropsychol Dev Cogn B Aging Neuropsychol Cogn 21: 146-173.

25. Lampit A, Hallock H, Moss R, Kwok S, Rosser M, et al. (2014) The timecourse of global cognitive gains from supervised computer-assisted cognitive training: A randomised, active-controlled trial in elderly with multiple dementia risk factors. J Prev Alz Dis 1: 33-39.

26. Lee Y, Lee C-R, Hwang B (2012) Effects of Computer-aided Cognitive Rehabilitation Training and Balance Exercise on Cognitive and Visual Perception Ability of the Elderly. Journal of Physical Therapy Science 24: 885-887.

27. Legault C, Jennings JM, Katula JA, Dagenbach D, Gaussoin SA, et al. (2011) Designing clinical trials for assessing the effects of cognitive training and physical activity interventions on cognitive outcomes: the Seniors Health and Activity Research Program Pilot (SHARP-P) study, a randomized controlled trial. BMC geriatrics 11: 27.

28. Li KZ, Roudaia E, Lussier M, Bherer L, Leroux A, et al. (2010) Benefits of cognitive dual-task training on balance performance in healthy older adults. J Gerontol A Biol Sci Med Sci 65: 1344-1352.

29. Lussier M, Gagnon C, Bherer L (2012) An investigation of response and stimulus modality transfer effects after dual-task training in younger and older. Front Hum Neurosci 6: 129.

30. Mahncke HW, Connor BB, Appelman J, Ahsanuddin ON, Hardy JL, et al. (2006) Memory enhancement in healthy older adults using a brain plasticity-based training program: a randomized, controlled study. Proc Natl Acad Sci U S A 103: 12523-12528.

31. Maillot P, Perrot A, Hartley A (2012) Effects of interactive physical-activity video-game training on physical and cognitive function in older adults. Psychol Aging 27: 589-600.

32. Mayas J, Parmentier FBR, Andres P, Ballesteros S (2014) Plasticity of attentional functions in older adults after non-action video game training: A randomized controlled trial. PLoS ONE 9.

33. McAvinue LP, Golemme M, Castorina M, Tatti E, Pigni FM, et al. (2013) An evaluation of a working memory training scheme in older adults. Front Aging Neurosci 5: 20.

34. Miller KJ, Dye RV, Kim J, Jennings JL, O'Toole E, et al. (2013) Effect of a computerized brain exercise program on cognitive performance in older adults. Am J Geriatr Psychiatry 21: 655-663.

35. Nouchi R, Taki Y, Takeuchi H, Hashizume H, Akitsuki Y, et al. (2012) Brain training game improves executive functions and processing speed in the elderly: a randomized controlled trial. PLoS One 7: e29676.

36. O'Brien JL, Edwards JD, Maxfield ND, Peronto CL, Williams VA, et al. (2013) Cognitive training and selective attention in the aging brain: An electrophysiological study. Clin Neurophysiol 124: 2198-2208.

37. Peng H, Wen J, Wang D, Gao Y (2012) The impact of processing speed training on working memory in old adults. Journal of Adult Development 19: 150-157.

38. Peretz C, Korczyn AD, Shatil E, Aharonson V, Birnboim S, et al. (2011) Computer-based, personalized cognitive training versus classical computer games: A randomized double-blind prospective trial of cognitive stimulation. Neuroepidemiology 36: 91-99.

39. Rasmusson DX, Rebok GW, Bylsma FW, Brandt J (1999) Effects of three types of memory training in normal elderly. Aging, Neuropsychology, and Cognition 6: 56-66.

40. Richmond LL, Morrison AB, Chein JM, Olson IR (2011) Working memory training and transfer in older adults. Psychol Aging 26: 813-822.

41. Sandberg P, Ronnlund M, Nyberg L, Stigsdotter Neely A (2014) Executive process training in young and old adults. Neuropsychol Dev Cogn B Aging Neuropsychol Cogn 21: 577-605.

42. Shatil E (2013) Does combined cognitive training and physical activity training enhance cognitive abilities more than either alone? A four-condition randomized controlled trial among healthy older adults. Front Aging Neurosci 5: 8.

43. Shatil E, Mikulecka J, Bellotti F, Bures V (2014) Novel television-based cognitive training improves working memory and executive function. PLoS One 9: e101472.

44. Simpson T, Camfield D, Pipingas A, Macpherson H, Stough C (2012) Improved processing speed: Online computer-based cognitive training in older adults. Educational Gerontology 38: 445-458.

45. Smith GE, Housen P, Yaffe K, Ruff R, Kennison RF, et al. (2009) A cognitive training program based on principles of brain plasticity: results from the Improvement in Memory with Plasticity-based Adaptive Cognitive Training (IMPACT) study. J Am Geriatr Soc 57: 594-603.

46. Stern Y, Blumen HM, Rich LW, Richards A, Herzberg G, et al. (2011) Space Fortress game training and executive control in older adults: a pilot intervention. Neuropsychol Dev Cogn B Aging Neuropsychol Cogn 18: 653-677.

47. van Muijden J, Band GP, Hommel B (2012) Online games training aging brains: limited transfer to cognitive control functions. Front Hum Neurosci 6: 221.

48. Vance D, Dawson J, Wadley V, Edwards J, Roenker D, et al. (2007) The accelerate study: The longitudinal effect of speed of processing training on cognitive performance of older adults. Rehabilitation Psychology 52: 89-96.

49. von Bastian CC, Langer N, Jancke L, Oberauer K (2013) Effects of working memory training in young and old adults. Mem Cognit 41: 611-624.

50. Wang MY, Chang CY, Su SY (2011) What's Cooking? - Cognitive Training of Executive Function in the Elderly. Front Psychol 2: 228.

51. Wolinsky FD, Vander Weg MW, Howren MB, Jones MP, Martin R, et al. (2011) Interim analyses from a randomised controlled trial to improve visual processing speed in older adults: the Iowa Healthy and Active Minds Study. BMJ Open 1: e000225.
