# Supplementary material for: Computerized Cognitive Training in Cognitively Healthy Older Adults: A Systematic Review and Meta-Analysis of Effect Modifiers
Source: PLoS Med. 2014 Nov 18;11(11):e1001756. doi: 10.1371/journal.pmed.1001756 (PMC4236015; doi:10.1371/journal.pmed.1001756)
Supplement: Table S3 — Data provided by primary authors. (DOCX) [file pmed.1001756.s011.docx]

**Table S3: Data provided by primary authors**

| Study | Data provided | Comments |
| --- | --- | --- |
| Anguera 2013[1] | Group-level means and SD for all transfer measures | The original article reported effect sizes and statistical tests |
| Ball 2002[2] | Patient-level results for transfer measures (SOP and control groups) | The original article reported composite measures, effect sizes and statistical tests |
| Barnes 2013[3] | Group-level means and SD for all transfer measures | The original article reported effect sizes and statistical tests |
| Berry 2010[4] | Group-level means and SD for all transfer measures | The original article reported statistical tests; change scores were provided in figures |
| Bottiroli 2009[5] | Group-level means and SD for all transfer measures | The original article reported statistical tests; raw scores were provided in figures |
| Garcia-Campuzano 2013[5] | Patient-level means and SD for WMS components | The original article reported only WMS factor scores |
| Lampit 2014[6] | Patient-level results for transfer measures | The study was conducted by the authors of this review. The original article reported effect sizes and statistical tests |
| Li 2010[7] | Group-level means and SD for all transfer measures | The original article did not report cognitive outcomes |
| Lussier 2012[8] | Group-level means and SD for all transfer measures | The original article reported effect sizes and statistical tests |
| Maillot 2012[9] | Group-level means and SD for all transfer measures | The original article reported change scores (mean and SD of difference from baseline to post-training) and statistical tests |
| Mayas 2014[10] | Group-level means and SD for the attention measures | Other cognitive outcomes from this study are reported in a separate paper that was still under review by the time this review was finalized |
| O'Brien 2013[11] | Patient-level results for all transfer measures | The original article reported only statistical tests |
| Peng 2012[12] | Group-level means and SD for all transfer measures | The original article reported effect sizes and statistical tests; raw scores were provided in figures |
| van Muijden 2012 [13] | Patient-level UFOV 2+3 results | Comparable to group-level data reported in the paper; data for UFOV 1 and total scores were not abailbles. |

**References**

1. Anguera JA, Boccanfuso J, Rintoul JL, Al-Hashimi O, Faraji F, et al. (2013) Video game training enhances cognitive control in older adults. Nature 501: 97-101.

2. Ball K, Berch DB, Helmers KF, Jobe JB, Leveck MD, et al. (2002) Effects of cognitive training interventions with older adults: a randomized controlled trial. Jama 288: 2271-2281.

3. Barnes DE, Santos-Modesitt W, Poelke G, Kramer AF, Castro C, et al. (2013) The Mental Activity and eXercise (MAX) trial: a randomized controlled trial to enhance cognitive function in older adults. JAMA Intern Med 173: 797-804.

4. Berry AS, Zanto TP, Clapp WC, Hardy JL, Delahunt PB, et al. (2010) The influence of perceptual training on working memory in older adults. PLoS One 5: e11537.

5. Bottiroli S, Cavallini E (2009) Can computer familiarity regulate the benefits of computer-based memory training in normal aging? A study with an Italian sample of older adults. Neuropsychol Dev Cogn B Aging Neuropsychol Cogn 16: 401-418.

6. Lampit A, Hallock H, Moss R, Kwok S, Rosser M, et al. (2014) The timecourse of global cognitive gains from supervised computer-assisted cognitive training: A randomised, active-controlled trial in elderly with multiple dementia risk factors. J Prev Alz Dis 1: 33-39.

7. Li KZ, Roudaia E, Lussier M, Bherer L, Leroux A, et al. (2010) Benefits of cognitive dual-task training on balance performance in healthy older adults. J Gerontol A Biol Sci Med Sci 65: 1344-1352.

8. Lussier M, Gagnon C, Bherer L (2012) An investigation of response and stimulus modality transfer effects after dual-task training in younger and older. Front Hum Neurosci 6: 129.

9. Maillot P, Perrot A, Hartley A (2012) Effects of interactive physical-activity video-game training on physical and cognitive function in older adults. Psychol Aging 27: 589-600.

10. Mayas J, Parmentier FBR, Andres P, Ballesteros S (2014) Plasticity of attentional functions in older adults after non-action video game training: A randomized controlled trial. PLoS ONE 9.

11. O'Brien JL, Edwards JD, Maxfield ND, Peronto CL, Williams VA, et al. (2013) Cognitive training and selective attention in the aging brain: An electrophysiological study. Clin Neurophysiol 124: 2198-2208.

12. Peng H, Wen J, Wang D, Gao Y (2012) The impact of processing speed training on working memory in old adults. Journal of Adult Development 19: 150-157.

13. van Muijden J, Band GP, Hommel B (2012) Online games training aging brains: limited transfer to cognitive control functions. Front Hum Neurosci 6: 221.
