# Supplementary material for: Computerized Cognitive Training in Cognitively Healthy Older Adults: A Systematic Review and Meta-Analysis of Effect Modifiers
Source: PLoS Med. 2014 Nov 18;11(11):e1001756. doi: 10.1371/journal.pmed.1001756 (PMC4236015; doi:10.1371/journal.pmed.1001756)
Supplement: Table S4 — Risk of bias within studies. (DOCX) [file pmed.1001756.s012.docx]

Table S4: Risk of bias within studies

| Study | Sequence generation | Allocation concealment | Assessor blinding | Incomplete outcome data | Selective  outcome reporting | Other sources of bias | Summary^a^ |
| --- | --- | --- | --- | --- | --- | --- | --- |
| Ackerman 2010[[1](#_ENREF_1)] | Unclear | Unclear | Low | Low | Low | Low | **Low** |
| Anderson 2013[[2](#_ENREF_2)] | Unclear | Unclear | Low | High | Unclear | Low | **High** |
| Anguera 2013[[3](#_ENREF_3)] | Unclear | Unclear | High | High | Low | Low | **High** |
| Ball 2002[[4](#_ENREF_4)] | Unclear | Low | Low | Low | Low | Low | **Low** |
| Barnes 2013[[5](#_ENREF_5)] | Low | Low | Low | Low | Low | Low | **Low** |
| Basak 2008[[6](#_ENREF_6)] | Unclear | Unclear | Unclear | Low | Low | Low | **High** |
| Belchior 2013[[7](#_ENREF_7)] | Unclear | Unclear | Unclear | Low | Low | Low | **High** |
| Berry 2010[[8](#_ENREF_8)] | Unclear | Unclear | Low | Low | Low | Low | **Low** |
| Boot 2013[[9](#_ENREF_9)] | Unclear | Unclear | Low | High | Low | Low | **High** |
| Bottiroli 2009[[10](#_ENREF_10)] | Unclear | Unclear | High | Low | Low | Low | **High** |
| Bozoki 2013[[11](#_ENREF_11)] | Unclear | Unclear | Low | Low | Unclear | Low | **Low** |
| Brehmer 2012[[12](#_ENREF_12)] | Unclear | Unclear | High | High | Low | Low | **High** |
| Burki 2014[[13](#_ENREF_13)] | Low | Unclear | High | Low | Low | Low | **High** |
| Buschkuehl 2008[[14](#_ENREF_14)] | Low | Unclear | High | High | Low | Low | **High** |
| Casutt 2014[[15](#_ENREF_15)] | Unclear | Unclear | Low | High | High | Low | **High** |
| Colzato 2011[[16](#_ENREF_16)] | Unclear | Unclear | Low | High | Unclear | Low | **High** |
| Dahlin 2008[[17](#_ENREF_17)] | Unclear | Unclear | Unclear | High | Low | Low | **High** |
| Dustman 1992[[18](#_ENREF_18)] | Unclear | Unclear | Unclear | Unclear | Unclear | Low | **High** |
| Edwards 2002[[19](#_ENREF_19)] | Unclear | Unclear | High | Low | Low | Low | **High** |
| Edwards 2005[[20](#_ENREF_20)] | Unclear | Unclear | Low | Low | Low | Low | **Low** |
| Edwards 2013[[21](#_ENREF_21)] | Unclear | Unclear | High | High | Low | Low | **High** |
| Garcia-Campuzano 2013[[22](#_ENREF_22)] | Unclear | Unclear | Low | Low | Unclear | Low | **Low** |
| Goldstein 1997[[23](#_ENREF_23)] | Unclear | Unclear | High | Unclear | Unclear | Low | **High** |
| Heinzel 2013[[24](#_ENREF_24)] | Unclear | Unclear | high | Low | Low | Low | **High** |
| Lampit 2014[[25](#_ENREF_25)] | Low | Low | Low | Low | Low | High | **Low** |
| Lee 2012[[26](#_ENREF_26)] | Unclear | Unclear | Unclear | Unclear | Unclear | Low | **High** |
| Legault 2011[[27](#_ENREF_27)] | Low | Unclear | Unclear | Low | Low | Low | **High** |
| Li 2010[[28](#_ENREF_28)] | Unclear | Unclear | Low | Low | High | Low | **Low** |
| Lussier 2012[[29](#_ENREF_29)] | Unclear | Unclear | high | Unclear | Low | Low | **High** |
| Mahncke 2006[[30](#_ENREF_30)] | Unclear | Unclear | Low | High | High | Low | **High** |
| Maillot 2012[[31](#_ENREF_31)] | Unclear | Unclear | Low | Low | Unclear | Low | **Low** |
| Mayas 2014[[32](#_ENREF_32)] | Unclear | Unclear | High | High | High | Low | **High** |
| McAvinue 2013[[33](#_ENREF_33)] | Low | Low | Low | High | Low | Low | **High** |
| Miller 2013[[34](#_ENREF_34)] | Unclear | Unclear | Unclear | High | Unclear | Low | **High** |
| Nouchi 2012[[35](#_ENREF_35)] | Low | Unclear | Low | Low | Low | Low | **Low** |
| O’Brien 2013[[36](#_ENREF_36)] | Unclear | Unclear | Low | Low | Unclear | Low | **Low** |
| Peng 2012[[37](#_ENREF_37)] | Unclear | Unclear | High | Low | Unclear | Low | **High** |
| Peretz 2011[[38](#_ENREF_38)] | Low | Low | Low | Low | Low | Low | **Low** |
| Rasmusson 1999[[39](#_ENREF_39)] | Unclear | Unclear | Low | Low | Low | Low | **Low** |
| Richmond 2011[[40](#_ENREF_40)] | Unclear | Unclear | High | High | Low | Low | **High** |
| Sandberg 2014[[41](#_ENREF_41)] | Unclear | Unclear | Low | High | Low | Low | **High** |
| Shatil 2013[[42](#_ENREF_42)] | Unclear | Unclear | High | High | Low | Low | **High** |
| Shatil 2014[[43](#_ENREF_43)] | Unclear | High | High | Unclear | Low | Low | **High** |
| Simpson 2012[[44](#_ENREF_44)] | Unclear | Low | Unclear | High | Low | Low | **High** |
| Smith 2009[[45](#_ENREF_45)] | Low | Low | Low | Low | Low | Low | **Low** |
| Stern 2011[[46](#_ENREF_46)] | Low | Unclear | High | High | Low | Low | **High** |
| van Muijden 2012[[47](#_ENREF_47)] | Unclear | Unclear | high | High | Low | Low | **High** |
| Vance et al 2007[[48](#_ENREF_48)] | Unclear | Unclear | Low | Low | Low | Low | **Low** |
| von Bastian 2013[[49](#_ENREF_49)] | Unclear | Unclear | Low | Low | Low | Low | **Low** |
| Wang 2011[[50](#_ENREF_50)] | Unclear | Unclear | High | High | Unclear | Low | **High** |
| Wolinsky 2011[[51](#_ENREF_51)] | Low | Low | Low | Low | Low | Low | **Low** |

Note. ^a^ We considered trials with high or unclear risk of bias those that did not include assessor blinding or did not perform intention-to-treat analyses. We considered all other trials as being at low risk of bias.

**References**

1. Ackerman PL, Kanfer R, Calderwood C (2010) Use it or lose it? Wii brain exercise practice and reading for domain knowledge. Psychol Aging 25: 753-766.

2. Anderson S, White-Schwoch T, Parbery-Clark A, Kraus N (2013) Reversal of age-related neural timing delays with training. Proc Natl Acad Sci U S A 110: 4357-4362.

3. Anguera JA, Boccanfuso J, Rintoul JL, Al-Hashimi O, Faraji F, et al. (2013) Video game training enhances cognitive control in older adults. Nature 501: 97-101.

4. Ball K, Berch DB, Helmers KF, Jobe JB, Leveck MD, et al. (2002) Effects of cognitive training interventions with older adults: a randomized controlled trial. Jama 288: 2271-2281.

5. Barnes DE, Santos-Modesitt W, Poelke G, Kramer AF, Castro C, et al. (2013) The Mental Activity and eXercise (MAX) trial: a randomized controlled trial to enhance cognitive function in older adults. JAMA Intern Med 173: 797-804.

6. Basak C, Boot WR, Voss MW, Kramer AF (2008) Can training in a real-time strategy video game attenuate cognitive decline in older adults? Psychol Aging 23: 765-777.

7. Belchior P, Marsiske M, Sisco SM, Yam A, Bavelier D, et al. (2013) Video game training to improve selective visual attention in older adults. Comput Human Behav 29: 1318-1324.

8. Berry AS, Zanto TP, Clapp WC, Hardy JL, Delahunt PB, et al. (2010) The influence of perceptual training on working memory in older adults. PLoS One 5: e11537.

9. Boot WR, Champion M, Blakely DP, Wright T, Souders DJ, et al. (2013) Video games as a means to reduce age-related cognitive decline: attitudes, compliance, and effectiveness. Front Psychol 4: 31.

10. Bottiroli S, Cavallini E (2009) Can computer familiarity regulate the benefits of computer-based memory training in normal aging? A study with an Italian sample of older adults. Neuropsychol Dev Cogn B Aging Neuropsychol Cogn 16: 401-418.

11. Bozoki A, Radovanovic M, Winn B, Heeter C, Anthony JC (2013) Effects of a computer-based cognitive exercise program on age-related cognitive decline. Arch Gerontol Geriatr 57: 1-7.

12. Brehmer Y, Westerberg H, Backman L (2012) Working-memory training in younger and older adults: training gains, transfer, and maintenance. Front Hum Neurosci 6: 63.

13. Burki CN, Ludwig C, Chicherio C, de Ribaupierre A (2014) Individual differences in cognitive plasticity: an investigation of training curves in younger and older adults. Psychol Res.

14. Buschkuehl M, Jaeggi SM, Hutchison S, Perrig-Chiello P, Dapp C, et al. (2008) Impact of working memory training on memory performance in old-old adults. Psychol Aging 23: 743-753.

15. Casutt G, Theill N, Martin M, Keller M, Jancke L (2014) The drive-wise project: Driving simulator training increases real driving performance in healthy older drivers. Frontiers in Aging Neuroscience 6.

16. Colzato LS, van Muijden J, Band GP, Hommel B (2011) Genetic Modulation of Training and Transfer in Older Adults: BDNF ValMet Polymorphism is Associated with Wider Useful Field of View. Front Psychol 2: 199.

17. Dahlin E, Nyberg L, Backman L, Neely AS (2008) Plasticity of executive functioning in young and older adults: immediate training gains, transfer, and long-term maintenance. Psychol Aging 23: 720-730.

18. Dustman RE, Emmerson RY, Steinhaus LA, Shearer DE, Dustman TJ (1992) The effects of videogame playing on neuropsychological performance of elderly individuals. J Gerontol 47: P168-171.

19. Edwards JD, Wadley VG, Myers RS, Roenker DL, Cissell GM, et al. (2002) Transfer of a speed of processing intervention to near and far cognitive functions. Gerontology 48: 329-340.

20. Edwards JD, Wadley VG, Vance DE, Wood K, Roenker DL, et al. (2005) The impact of speed of processing training on cognitive and everyday performance. Aging Ment Health 9: 262-271.

21. Edwards JD, Valdes EG, Peronto C, Castora-Binkley M, Alwerdt J, et al. (2013) The Efficacy of InSight Cognitive Training to Improve Useful Field of View Performance: A Brief Report. J Gerontol B Psychol Sci Soc Sci.

22. Garcia-Campuzano MT, Virues-Ortega J, Smith S, Moussavi Z (2013) Effect of cognitive training targeting associative memory in the elderly: A small randomized trial and a longitudinal evaluation. Journal of the American Geriatrics Society 61: 2252-2254.

23. Goldstein J, Cajko L, Oosterbroek M, Michielsen M, Van Houten O, et al. (1997) Video Games and the Elderly. Social Behavior and Personality: an international journal 25: 345-352.

24. Heinzel S, Schulte S, Onken J, Duong QL, Riemer TG, et al. (2014) Working memory training improvements and gains in non-trained cognitive tasks in young and older adults. Neuropsychol Dev Cogn B Aging Neuropsychol Cogn 21: 146-173.

25. Lampit A, Hallock H, Moss R, Kwok S, Rosser M, et al. (2014) The timecourse of global cognitive gains from supervised computer-assisted cognitive training: A randomised, active-controlled trial in elderly with multiple dementia risk factors. J Prev Alz Dis 1: 33-39.

26. Lee Y, Lee C-R, Hwang B (2012) Effects of Computer-aided Cognitive Rehabilitation Training and Balance Exercise on Cognitive and Visual Perception Ability of the Elderly. Journal of Physical Therapy Science 24: 885-887.

27. Legault C, Jennings JM, Katula JA, Dagenbach D, Gaussoin SA, et al. (2011) Designing clinical trials for assessing the effects of cognitive training and physical activity interventions on cognitive outcomes: the Seniors Health and Activity Research Program Pilot (SHARP-P) study, a randomized controlled trial. BMC geriatrics 11: 27.

28. Li KZ, Roudaia E, Lussier M, Bherer L, Leroux A, et al. (2010) Benefits of cognitive dual-task training on balance performance in healthy older adults. J Gerontol A Biol Sci Med Sci 65: 1344-1352.

29. Lussier M, Gagnon C, Bherer L (2012) An investigation of response and stimulus modality transfer effects after dual-task training in younger and older. Front Hum Neurosci 6: 129.

30. Mahncke HW, Connor BB, Appelman J, Ahsanuddin ON, Hardy JL, et al. (2006) Memory enhancement in healthy older adults using a brain plasticity-based training program: a randomized, controlled study. Proc Natl Acad Sci U S A 103: 12523-12528.

31. Maillot P, Perrot A, Hartley A (2012) Effects of interactive physical-activity video-game training on physical and cognitive function in older adults. Psychol Aging 27: 589-600.

32. Mayas J, Parmentier FBR, Andres P, Ballesteros S (2014) Plasticity of attentional functions in older adults after non-action video game training: A randomized controlled trial. PLoS ONE 9.

33. McAvinue LP, Golemme M, Castorina M, Tatti E, Pigni FM, et al. (2013) An evaluation of a working memory training scheme in older adults. Front Aging Neurosci 5: 20.

34. Miller KJ, Dye RV, Kim J, Jennings JL, O'Toole E, et al. (2013) Effect of a computerized brain exercise program on cognitive performance in older adults. Am J Geriatr Psychiatry 21: 655-663.

35. Nouchi R, Taki Y, Takeuchi H, Hashizume H, Akitsuki Y, et al. (2012) Brain training game improves executive functions and processing speed in the elderly: a randomized controlled trial. PLoS One 7: e29676.

36. O'Brien JL, Edwards JD, Maxfield ND, Peronto CL, Williams VA, et al. (2013) Cognitive training and selective attention in the aging brain: An electrophysiological study. Clin Neurophysiol 124: 2198-2208.

37. Peng H, Wen J, Wang D, Gao Y (2012) The impact of processing speed training on working memory in old adults. Journal of Adult Development 19: 150-157.

38. Peretz C, Korczyn AD, Shatil E, Aharonson V, Birnboim S, et al. (2011) Computer-based, personalized cognitive training versus classical computer games: A randomized double-blind prospective trial of cognitive stimulation. Neuroepidemiology 36: 91-99.

39. Rasmusson DX, Rebok GW, Bylsma FW, Brandt J (1999) Effects of three types of memory training in normal elderly. Aging, Neuropsychology, and Cognition 6: 56-66.

40. Richmond LL, Morrison AB, Chein JM, Olson IR (2011) Working memory training and transfer in older adults. Psychol Aging 26: 813-822.

41. Sandberg P, Ronnlund M, Nyberg L, Stigsdotter Neely A (2014) Executive process training in young and old adults. Neuropsychol Dev Cogn B Aging Neuropsychol Cogn 21: 577-605.

42. Shatil E (2013) Does combined cognitive training and physical activity training enhance cognitive abilities more than either alone? A four-condition randomized controlled trial among healthy older adults. Front Aging Neurosci 5: 8.

43. Shatil E, Mikulecka J, Bellotti F, Bures V (2014) Novel television-based cognitive training improves working memory and executive function. PLoS One 9: e101472.

44. Simpson T, Camfield D, Pipingas A, Macpherson H, Stough C (2012) Improved processing speed: Online computer-based cognitive training in older adults. Educational Gerontology 38: 445-458.

45. Smith GE, Housen P, Yaffe K, Ruff R, Kennison RF, et al. (2009) A cognitive training program based on principles of brain plasticity: results from the Improvement in Memory with Plasticity-based Adaptive Cognitive Training (IMPACT) study. J Am Geriatr Soc 57: 594-603.

46. Stern Y, Blumen HM, Rich LW, Richards A, Herzberg G, et al. (2011) Space Fortress game training and executive control in older adults: a pilot intervention. Neuropsychol Dev Cogn B Aging Neuropsychol Cogn 18: 653-677.

47. van Muijden J, Band GP, Hommel B (2012) Online games training aging brains: limited transfer to cognitive control functions. Front Hum Neurosci 6: 221.

48. Vance D, Dawson J, Wadley V, Edwards J, Roenker D, et al. (2007) The accelerate study: The longitudinal effect of speed of processing training on cognitive performance of older adults. Rehabilitation Psychology 52: 89-96.

49. von Bastian CC, Langer N, Jancke L, Oberauer K (2013) Effects of working memory training in young and old adults. Mem Cognit 41: 611-624.

50. Wang MY, Chang CY, Su SY (2011) What's Cooking? - Cognitive Training of Executive Function in the Elderly. Front Psychol 2: 228.

51. Wolinsky FD, Vander Weg MW, Howren MB, Jones MP, Martin R, et al. (2011) Interim analyses from a randomised controlled trial to improve visual processing speed in older adults: the Iowa Healthy and Active Minds Study. BMJ Open 1: e000225.
