# Supplementary material for: Computerized Cognitive Training in Cognitively Healthy Older Adults: A Systematic Review and Meta-Analysis of Effect Modifiers
Source: PLoS Med. 2014 Nov 18;11(11):e1001756. doi: 10.1371/journal.pmed.1001756 (PMC4236015; doi:10.1371/journal.pmed.1001756)
Supplement: Table S5 — Results of sensitivity analyses. (DOCX) [file pmed.1001756.s013.docx]

**Table S5: Sensitivity analyses (k=51)**

| **Analysis** | **Model** | **Within-study multiple outcome correlation** | **Pre-post correlation** | **Hedges’ *g* (95% CI)** | ***I^2^*, %(95% CI)** |
| --- | --- | --- | --- | --- | --- |
| **Fixed vs random-effects model** | ***Random*** | ***0.7*** | ***0.6*** | ***0.22 (0.15 to 0.29)*** | ***29.92 (0.63 to 50.57)*** |
|  | Fixed | 0.7 | 0.6 | 0.20 (0.15 to 0.25) | 29.92 (0.63 to 50.57) |
| **Within-study multiple outcome correlation** | Random | 0.6 | 0.6 | 0.22 (0.16 to 0.30) | 36.80 (11.02 to 55.11) |
|  | Random | 0.8 | 0.6 | 0.22 (0.15 to 0.30) | 23.55 (0 to 46.34) |
| **Pre-post correlation** | Random | 0.7 | 0.5 | 0.20 (0.14 to 0.27) | 15.97 (0 to 41.12) |
|  | Random | 0.7 | 0.7 | 0.26 (0.18 to 0.34) | 44.80 (23.07 to 60.39) |
| **Inclusion of passive instead of active control groups in 3 studies** | Random | 0.7 | 0.6 | 0.22 (0.15 to 0.29) | 28.96 (0 to 49.94) |
