# Supplementary material for: Computerized Cognitive Training in Cognitively Healthy Older Adults: A Systematic Review and Meta-Analysis of Effect Modifiers
Source: PLoS Med. 2014 Nov 18;11(11):e1001756. doi: 10.1371/journal.pmed.1001756 (PMC4236015; doi:10.1371/journal.pmed.1001756)
Supplement: Protocol S1 — Study protocol. (DOCX) [file pmed.1001756.s016.docx]

Protocol S1:

**Computerised Cognitive Training in Healthy Older Adults: A Systematic Review and Meta-Analysis of Effect Modifiers**

**Review team:** A/Prof Michael Valenzuela, Dr Amit Lampit, Mr Hariharan Hallock.

**Students:** Ms Bridget Dijkmans-Hadley, Ms Rebecca Moss, Ms Anna Radowiecka.

# Rationale

Human adult ageing is associated with a gradual process of age-related cognitive decline (ARCD). Further deterioration in cognition can lead to mild cognitive decline (MCI) and dementia, whose prevalence in adults over 65 years of age is estimated at 20% and 15%, respectively [[1](#_ENREF_1)]. Given strong links between engagement in cognitively stimulating activities and enhanced late-life cognition and reduced risk of MCI and dementia [[2-4](#_ENREF_2)], there has been growing interest in cognition-focused interventions that may attenuate ARCD and help maintain cognitive performance in older adults. Arguably, the most studied intervention is cognitive training (CT), which involves structured practice on standardised cognitively challenging tasks [[5](#_ENREF_5)]. Despite a wealth of studies linking CT to various cognitive benefits and neuroplastic changes in older adults [[6-9](#_ENREF_6)], the current evidence base is methodologically heterogeneous and results inconsistent [[5](#_ENREF_5)].

Recent years has seen a sharp increase in the popularity of computer-assisted cognitive training (CCT) among researchers and the public alike. CCT has several advantages over traditional CT methods, including more visually appealing interfaces, efficient and scalable delivery and the ability to constantly adapt training content and difficulty to individual performance [[6](#_ENREF_6),[10](#_ENREF_10),[11](#_ENREF_11)]. The CCT literature in healthy elderly has been criticised for lack of adequate control groups, poor study designs, and inconsistent methodology in terms of program design, dose and outcome measures. Whether these criticisms are valid generally has not yet been tested in a systematic review and quantitative meta-analysis. Rather, previous systematic reviews in this area have mixed CCT with non-CCT interventions, or mixed results from RCTs with non-RCT designs [[5-9](#_ENREF_5)] . Hence, the overall efficacy of CCT in the healthy aged is currently unclear and researchers and clinicians have no information about what design factors may influence outcome.

# Objectives

In healthy older adults, we aim to:

1. Evaluate the efficacy of randomised controlled trials (RCTs) of CCT on cognitive outcomes;
2. Test the moderating effects of several key design features;
3. Assess the nature and quality of RCT evidence; and
4. Suggest recommendations for future CCT research based on these findings.

# Eligibility criteria

The inclusion and exclusion criteria apply to whole studies or components from individual studies. That is, studies may meet inclusion criteria but parts of them (e.g., certain outcomes or study population) may meet exclusion criteria and therefore excluded from the review.

## Inclusion criteria

### Types of studies

Randomised controlled trials (RCTs) studying the effect(s) of CCT on one or more cognitive outcomes in healthy older adults. There will be no restrictions for date of publication or language.

### Types of participants

Mean participant age ≥60 years; lack of any known cognitive (including MCI and dementia of any aetiology), neurological (e.g., epilepsy, stroke), psychiatric (e.g., schizophrenia) and/or sensory impairment.

### Types of interventions

*CCT:* minimum 4 hours of practice on standardised computerised tasks with clear cognitive rationale [[5](#_ENREF_5),[10](#_ENREF_10)] or video games, administered on personal computers, mobile devices or gaming consoles. Video games are defined as computer programs that were distributed for entertainment purposes before they were tried as cognitive interventions [[12](#_ENREF_12)]. Studies combining CCT with other non-pharmacological interventions (e.g., physical exercise) and 2X2 designs will be eligible as long as the control group received the same adjuvant intervention.

*Control:* Any type of control condition will be eligible, including wait-list, no-contact and active (‘placebo’) control groups, except for alternative active treatments such as comparable paper-based version of the CCT exercises. Physical exercise as a control condition will included only when 1) the CCT group received the same exercise regimen, and 2) the control group received an active cognitive control intervention in addition to physical exercise.

### Types of outcome measures

Scores in cognitive tests that were not included in the training program (i.e., untrained), administered both before and after training, measuring change in performance in one or more of the following domains: global cognition, verbal memory, non-verbal memory, working memory, processing speed, attention, language/fluency, visuospatial skills and executive functions. All outcomes that meet this definition will be included in the review, including secondary outcomes and multiple tests of the same cognitive domain.

## Exclusion criteria

### Types of participants

Studies will be excluded if 1) their inclusion criteria are restricted to a specific neuropsychiatric population (such as MCI, dementia) or any type of neurological, psychiatric, behavioural and/or physical condition that may affect cognition (e.g., heart disease); 2) mean baseline scores indicate abnormally low cognitive performance in the sample. Studies that include both old and young adult groups will be included, but younger group data will be excluded from the review.

### Types of interventions

*CCT:* studies (or groups within studies) will not be included if: 1) more than 50% of the intervention was not conducted on a computer; 2) the intervention does not involve interaction with a computer (e.g., overhead projectors); 3) the intervention includes pharmacological intervention, brain stimulation or neurofeedback; or 4) the CCT group does not have a matching control group.

*Control:* Studies that compare CCT to physical exercise alone will be excluded.

### Types of outcomes

Outcomes will be excluded if they were: 1) not administered both at baseline and immediately after training; 2) long-term post-training outcomes; 3) subjective (e.g., questionnaires) or non-cognitive (e.g., mood, physical, neuroimaging); or 4) measuring activities of daily living rather than performance in cognitive tests.

# Search strategy

## Information sources

Potentially eligible published articles and data will be located by:

1. Electronic database search on Medline, Embase and PsychINFO;
2. Inspection of reference lists of previous reviews in the wider field of cognitive interventions [[5-11](#_ENREF_5),[13](#_ENREF_13)]; and
3. By contacting authors for unpublished cognitive outcomes.

## Search terms

Because there is no consistent terminology in the field, this review will conduct an extensive literature search that will cover various known terms that essentially describe CCT without any restrictions. These will be:

- cognitive training
- brain training
- memory training
- attention training
- reasoning training
- computerized training
- computer training
- video game
- computer game

# Study selection

AL will conduct the database search, download the results to a single Endnote library, remove duplicates and add papers identified from other sources. AL and HH will then independently screen all the results for initial eligibility based on title and abstract. The full text of potentially eligible studies and studies whose eligibility is unclear based on title and abstract will be assessed by AL and HH, who will also contact authors when eligibility is unclear based on the full-text article. Disagreements regarding study eligibility will be resolved by a third reviewer (MV).

# Data collection process

Cognitive outcome data will be extracted in the form of means and standard deviations of each group at baseline and follow-up only, and entered directly by HH into a spreadsheet developed for this review by AL. Missing raw data will be requested from study authors. Data in other formats (e.g., raw mean difference or effect sizes) should be extracted only if raw data is unavailable and the article provides sufficient information to reliably calculate standardised mean difference (SMD). Categorisation of each outcome to into cognitive domains will be performed by AL and HH by consulting The Compendium of Neuropsychological Tests [[14](#_ENREF_14)]. If the outcome is not discussed in the Compendium, categorisation will be performed by consensus among AL, HH and MV.

# Data items

The following information will be provided for each cognitive outcome:

1. Study name (e.g., Ball 2002)
2. Name of the test or section of a test (e.g. Trail Making Test B)
3. Raw data for each group (mean, SD, sample size) at baseline and follow-up
4. Cognitive domain (global cognition, verbal memory, non-verbal memory, working memory, processing speed, attention, language/fluency, visuospatial skills and executive functions)
5. Type of CCT program (e.g., working memory training)
6. Name of program (e.g., speed of processing training)
7. Delivery format (group or home-based training)
8. Total training duration (in hours)
9. Session length (in minutes)
10. Session frequency (sessions per week)
11. Control condition (active or passive control)
12. PEDro score (see below)
13. Risk of bias (high or low risk of bias, see below).

Additional study-level items will include sample size and characteristics (e.g., mean participant age) and country of origin.

# Risk of bias in individual studies

## Risk of bias assessment

Risk of bias in individual studies will be conducted in accordance with the Cochrane’s Collaboration’s risk of bias tool [[15](#_ENREF_15)], modified to the specific circumstances of CCT research. Low, high or unclear risk of bias will be determined for each of the following categories:

1. Sequence generation
2. Allocation concealment
3. Blinding of outcome assessors (blinding of personnel and participants will not be assessed)
4. Incomplete outcome data
5. Selective outcome reporting
6. Other sources of bias.

Trials with high or unclear risk of bias in items 3 and/or 4 will be considered as having high risk of bias. Assessments will be conducted independently by HH and an additional reviewer (BDH, RM or AR). HH or AL will contact authors if study reports do not provide insufficient details to determine risk of bias.

## Study quality

Quality of individual studies will be further assessed using an adapted version of the Physiotherapy Evidence Database (PEDro) scale [[16](#_ENREF_16)]. Assessments will be conducted independently by HH and an additional reviewer (BDH, RM or AR). Consensus score will be determined by AL. Items 5 (blinding of subjects) and 6 (blinding of therapists) will not be considered in the PEDro score.

# Summary measures

The primary outcome will be SMD (expressed as Hedges’s *g*) and 95% confidence intervals (CI) of post-training change between CCT and control groups. The analysis will be conducted for all cognitive outcomes combined and will be complemented by analyses of individual cognitive domains (global cognition, verbal memory, non-verbal memory, working memory, processing speed, attention, language, visuospatial skills and executive functions).

# Methods of analysis

All analyses will be using Comprehensive Meta Analysis (CMA, Biostat Inc., Englewood, NJ). To avoid selective analysis of outcomes, study-level SMDs from the same cognitive domain will be combined into a single effect estimate. Pooling of outcomes across studies will be conducted using random effects model. Heterogeneity across studies will be quantified using the *I*^2^ statistic [[17](#_ENREF_17)].

## Publication bias

Funnel plots will be visually inspected [[18](#_ENREF_18)] and formally tested using Egger’s Test of the Intercepts [[19](#_ENREF_19)]. If significant asymmetry will be detected (P<0.1) the meta-analysis for the cognitive outcome for which possible publication was found will be repeated using a fixed-effects model [[18](#_ENREF_18)].

## Additional analyses

In order to detect possible design factors that affect CCT efficacy we will perform subgroup meta-analyses. These analyses will be based on random-effects model, and between-subgroup heterogeneity will be tested using Cochrane’s Q (significant at the p<0.05 level). Analyses will be performed both overall and for each cognitive outcome based on the following items from section 7:

1. Type of CCT program (e.g., working memory training)
2. Delivery format (group or home-based training)
3. Total training duration (in hours)
4. Session length (in minutes)
5. Session frequency (sessions per week)
6. Control condition (active or passive control)
7. Risk of bias (high or low risk of bias, see below).

# References

1. Thies W, Bleiler L, Alzheimer's A (2013) 2013 Alzheimer's disease facts and figures. Alzheimers Dement 9: 208-245.

2. Marioni RE, Valenzuela MJ, van den Hout A, Brayne C, Matthews FE, et al. (2012) Active cognitive lifestyle is associated with positive cognitive health transitions and compression of morbidity from age sixty-five. PLoS One 7: e50940.

3. Verghese J, Lipton RB, Katz MJ, Hall CB, Derby CA, et al. (2003) Leisure activities and the risk of dementia in the elderly. N Engl J Med 348: 2508-2516.

4. Wilson RS, Mendes De Leon CF, Barnes LL, Schneider JA, Bienias JL, et al. (2002) Participation in cognitively stimulating activities and risk of incident Alzheimer disease. JAMA 287: 742-748.

5. Martin M, Clare L, Altgassen AM, Cameron MH, Zehnder F (2011) Cognition-based interventions for healthy older people and people with mild cognitive impairment. Cochrane Database Syst Rev: CD006220.

6. Kueider AM, Parisi JM, Gross AL, Rebok GW (2012) Computerized Cognitive Training with Older Adults: A Systematic Review. PLoS ONE 7: e40588.

7. Valenzuela M, Sachdev P (2009) Can cognitive exercise prevent the onset of dementia? Systematic review of randomized clinical trials with longitudinal follow-up. Am J Geriatr Psychiatry 17: 179-187.

8. Papp KV, Walsh SJ, Snyder PJ (2009) Immediate and delayed effects of cognitive interventions in healthy elderly: a review of current literature and future directions. Alzheimers Dement 5: 50-60.

9. Reijnders J, van Heugten C, van Boxtel M (2013) Cognitive interventions in healthy older adults and people with mild cognitive impairment: a systematic review. Ageing Res Rev 12: 263-275.

10. Gates N, Valenzuela M (2010) Cognitive exercise and its role in cognitive function in older adults. Curr Psychiatry Rep 12: 20-27.

11. Jak AJ, Seelye AM, Jurick SM (2013) Crosswords to computers: a critical review of popular approaches to cognitive enhancement. Neuropsychol Rev 23: 13-26.

12. Bavelier D, Green CS, Han DH, Renshaw PF, Merzenich MM, et al. (2011) Brains on video games. Nat Rev Neurosci 12: 763-768.

13. Buschert V, Bokde AL, Hampel H (2010) Cognitive intervention in Alzheimer disease. Nat Rev Neurol 6: 508-517.

14. Strauss EH, Sherman EMS, Spreen OA, editors (2006) A Compendium of Neuropsychological Tests: Administration, Norms and Commentary.: Oxford University Press.

15. Higgins J, Green S, editors (2011) Cochrane Handbook for Systematic Reviews of Interventions Version 5.1.0: The Cochrane Collaboration.

16. Maher CG, Sherrington C, Herbert RD, Moseley AM, Elkins M (2003) Reliability of the PEDro scale for rating quality of randomized controlled trials. Phys Ther 83: 713-721.

17. Higgins JP, Thompson SG, Deeks JJ, Altman DG (2003) Measuring inconsistency in meta-analyses. BMJ 327: 557-560.

18. Sterne JA, Sutton AJ, Ioannidis JP, Terrin N, Jones DR, et al. (2011) Recommendations for examining and interpreting funnel plot asymmetry in meta-analyses of randomised controlled trials. BMJ 343: d4002.

19. Egger M, Davey Smith G, Schneider M, Minder C (1997) Bias in meta-analysis detected by a simple, graphical test. BMJ 315: 629-634.
